# Supplementary material for: Machine learning-based tissue of origin classification for cancer of unknown primary diagnostics using genome-wide mutation features
Source: Nat Commun. 2022 Jul 11;13:4013. doi: 10.1038/s41467-022-31666-w (PMC9273599; doi:10.1038/s41467-022-31666-w)
Supplement: Supplementary file 1 — Supplementary information [file 41467_2022_31666_MOESM1_ESM.pdf]

Supplementary information

Supplementary figures

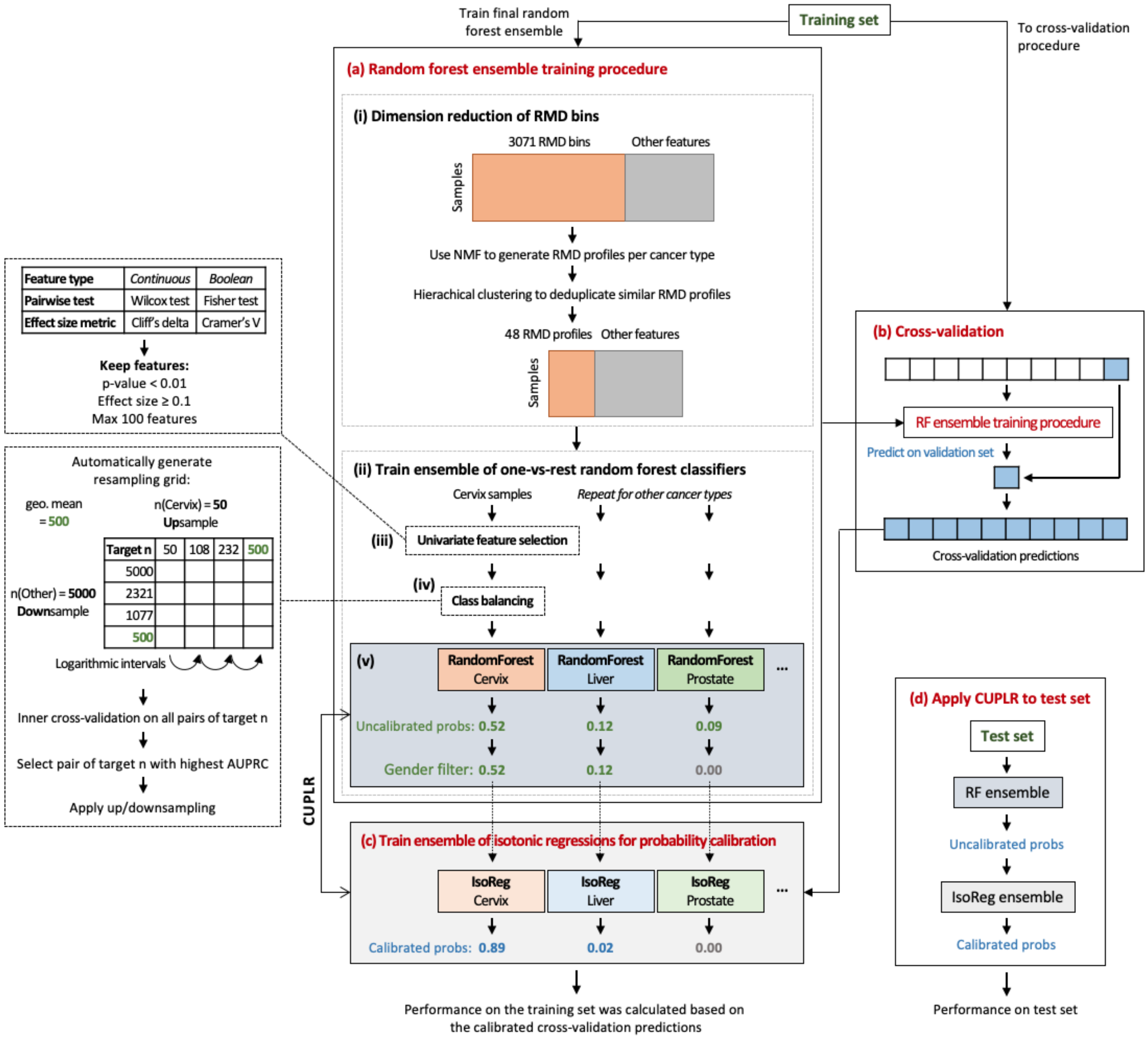

Supplementary figure 1: CUPLR training procedure. (a) An ensemble of binary random forest classifiers was trained each to discriminate one cancer type versus other cancer types. **(i)** Dimension reduction via non-negative matrix factorization (NMF) was performed on the 3071 RMD bins independently for each cancer type to ultimately produce 48 cancer type specific RMD profiles (see **Supplementary figure 2** for a detailed schematic; see **Supplementary data 6** for a visualization of each RMD profile), prior to random forest training as shown in **(ii)**. **(iii)** Univariate feature selection was performed to remove irrelevant features. (**iv**) Class resampling was performed to alleviate imbalances in the number of samples for each cancer type. (**v**) The random forests are trained. Breast, ovary and cervix probabilities from the random forest are set to 0 for male samples, and prostate probabilities are set to 0 for female samples. **(b)** The whole training procedure in **(a)** was subjected to 15-fold cross-validation to obtain cancer type probabilities for the training samples. **(c)** These probabilities were then used to train the isotonic regressions for probability calibration. The calibrated probabilities were also used to calculate cross-validation performance. **(d)** Performance was also determined by applying CUPLR to a held out test set.

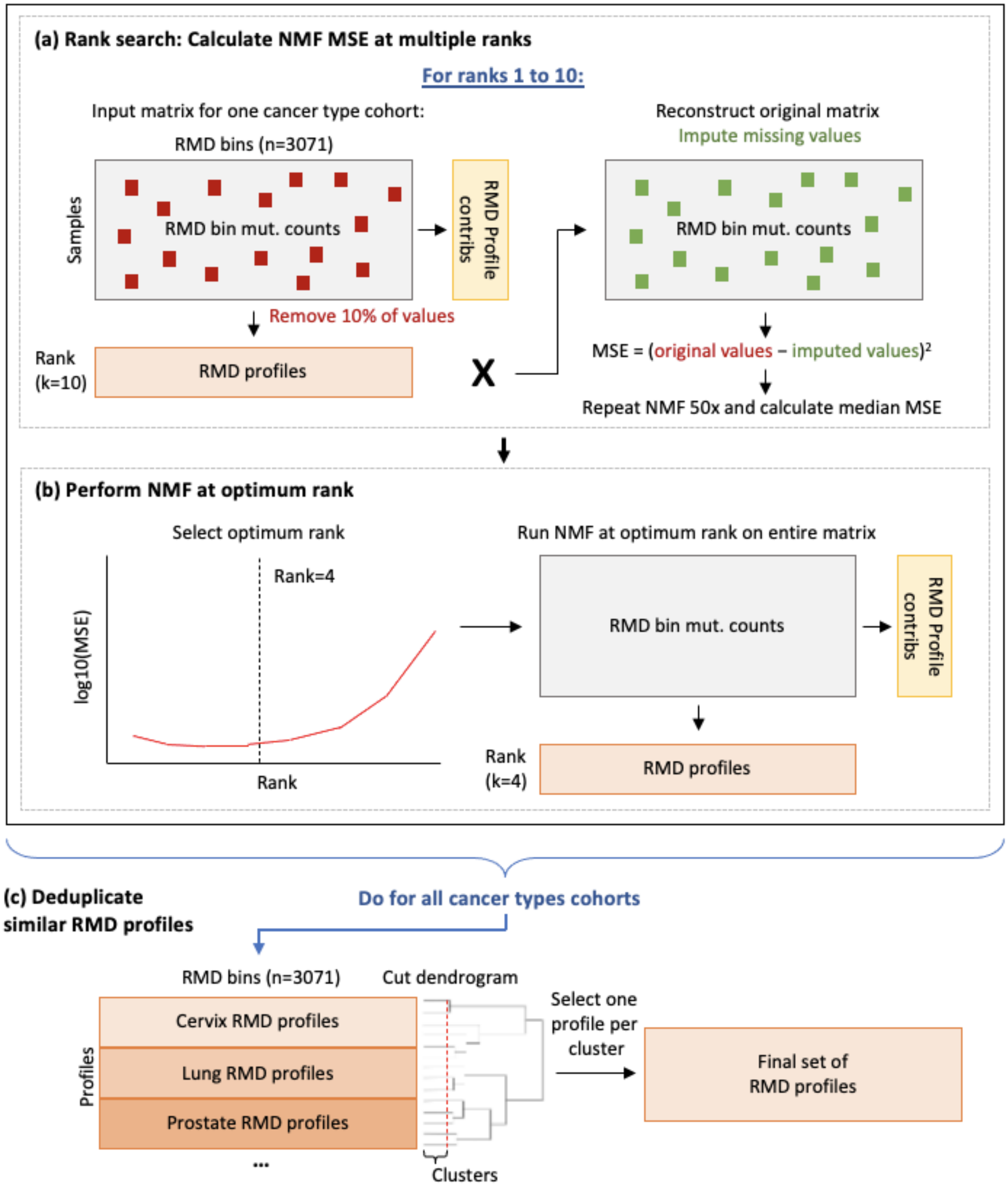

Supplementary figure 2: Extraction of regional mutational density (RMD) profiles. (a) Non-negative matrix factorization (NMF) was performed for several ranks to determine the optimum rank. For each rank, 10% of values were removed from the input matrix, NMF was performed, the original matrix was reconstructed, the missing values were imputed, and mean squared error between the original missing values and the imputed values. This was repeated 50 times and median MSE was calculated. **(b)** The optimum rank was the one at which the log10(MSE) value began to increase rapidly. NMF was performed on the entire input matrix (i.e. without removing values) to yield the RMD profiles for one cancer type cohort. **(c)** The procedures in (a) and (b) were performed for all cancer types to yield RMD profiles for all cancer types. Hierarchical clustering was performed to group profiles that were similar. One profile was selected per cluster to yield the final set of RMD profiles.

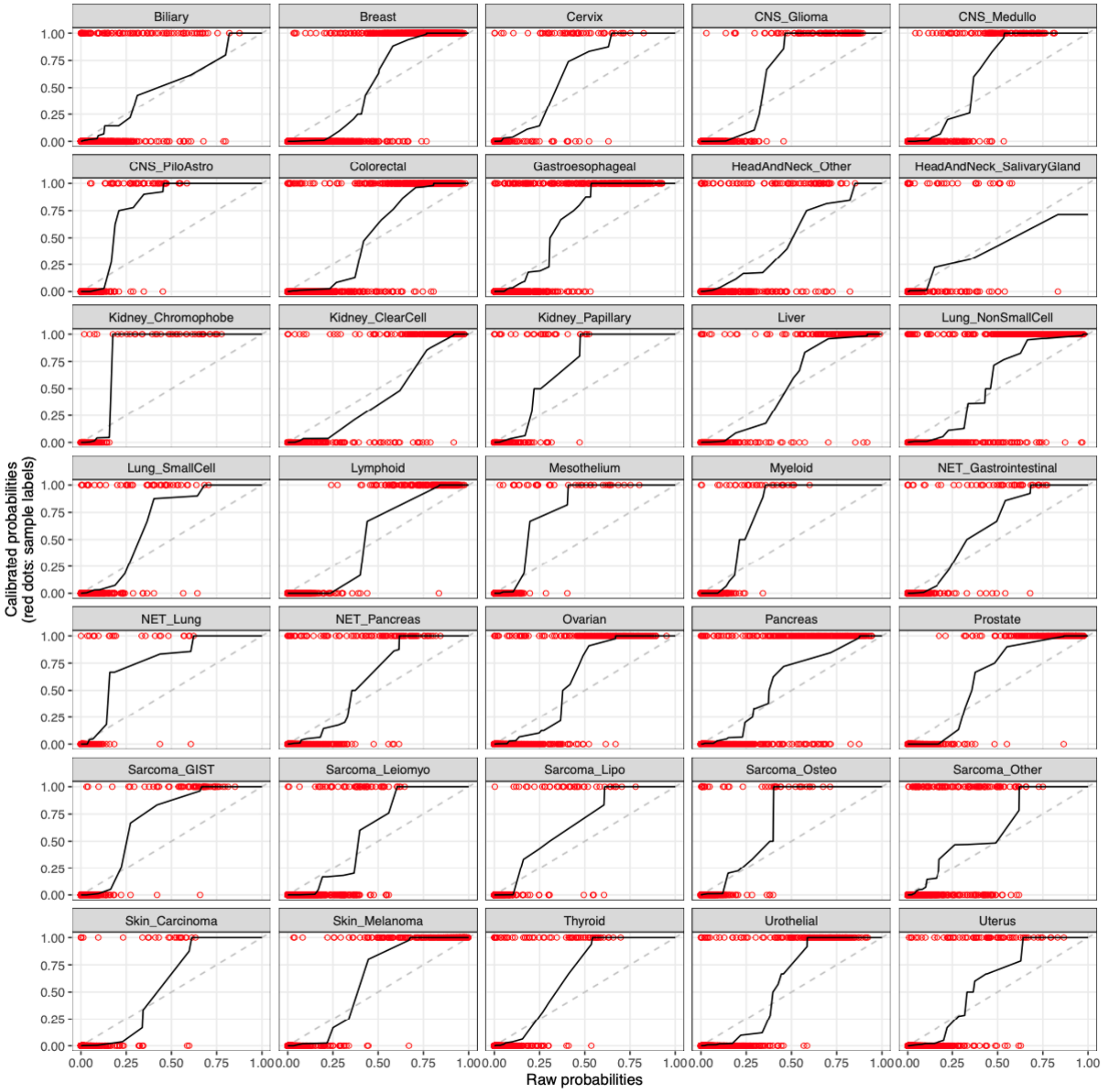

Supplementary figure 3: Isotonic regression calibration curves for each random forest in CUPLR. Red dots at y=1 are samples that were predicted by the respective cancer type random forest as that cancer type, whereas dots at y=0 are samples that were predicted as not being that cancer type. Cancer type predictions were obtained by cross-validation.

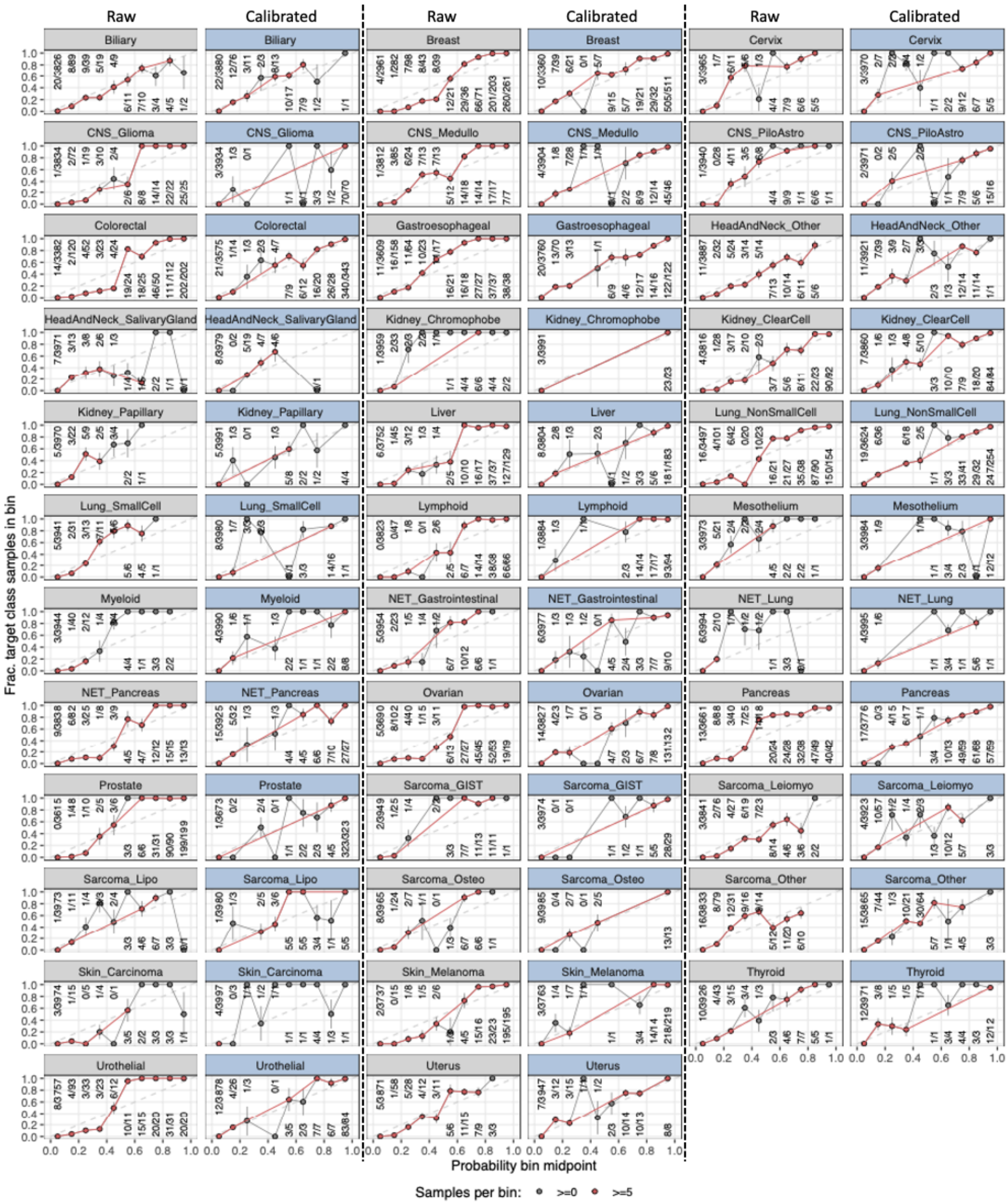

Supplementary figure 4: Reliability curves showing the probability biases before and after calibration. Grey panels show the curves before calibration and the blue panels show the curves after calibration. Each dot represents the fraction of samples of the target cancer type in a particular probability bin (a bin at e.g. 0.05 would represent probabilities between 0 and 0.1). Dots above the diagonal are probabilities where the random forest is overconfident whereas dots below the diagonal are probabilities where it is underconfident. A properly calibrated classifier has a reliability curve close to the diagonal. Two curves are shown in each panel, one filtered such that each bin has sufficient (≥5) samples for plotting a stable curve, and one where each bin has ≥0 samples representing the raw reliability curve.

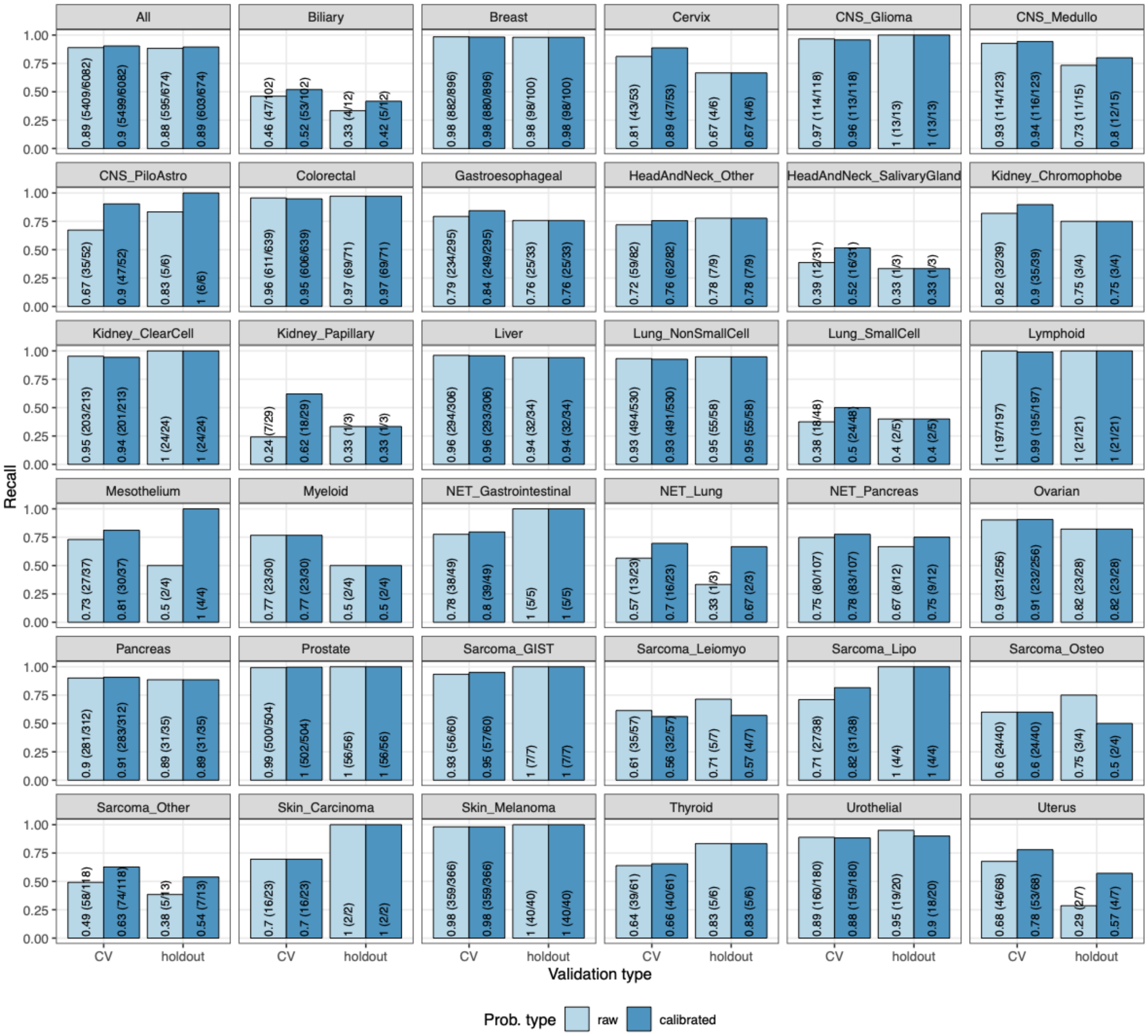

Supplementary figure 5: Performance of CUPLR before and after calibration of raw random forest probabilities. Recall (i.e. fraction of samples correctly predicted) was determined using cross validation on the training set (light blue bars) as well as by predicting on the held out test set (dark blue).

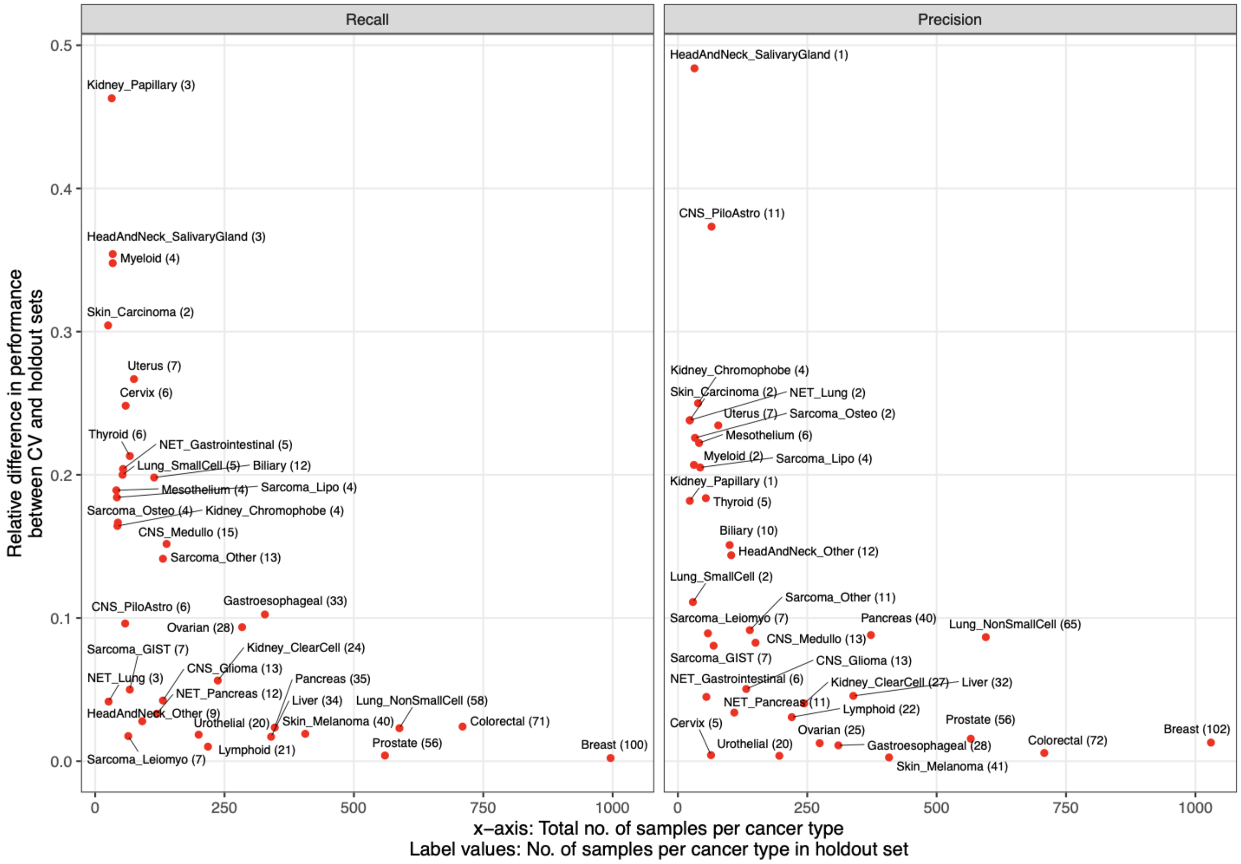

Supplementary figure 6: Relative difference in recall of CUPLR between the cross-validation (CV) predictions and predictions on the holdout test set. The relative difference ranges between 0 and 1 and was calculated using the formula: |a - b| / max(a, b); where a = CV performance and b = holdout performance. Small cancer type cohorts show higher variation between CV and holdout recall and precision.

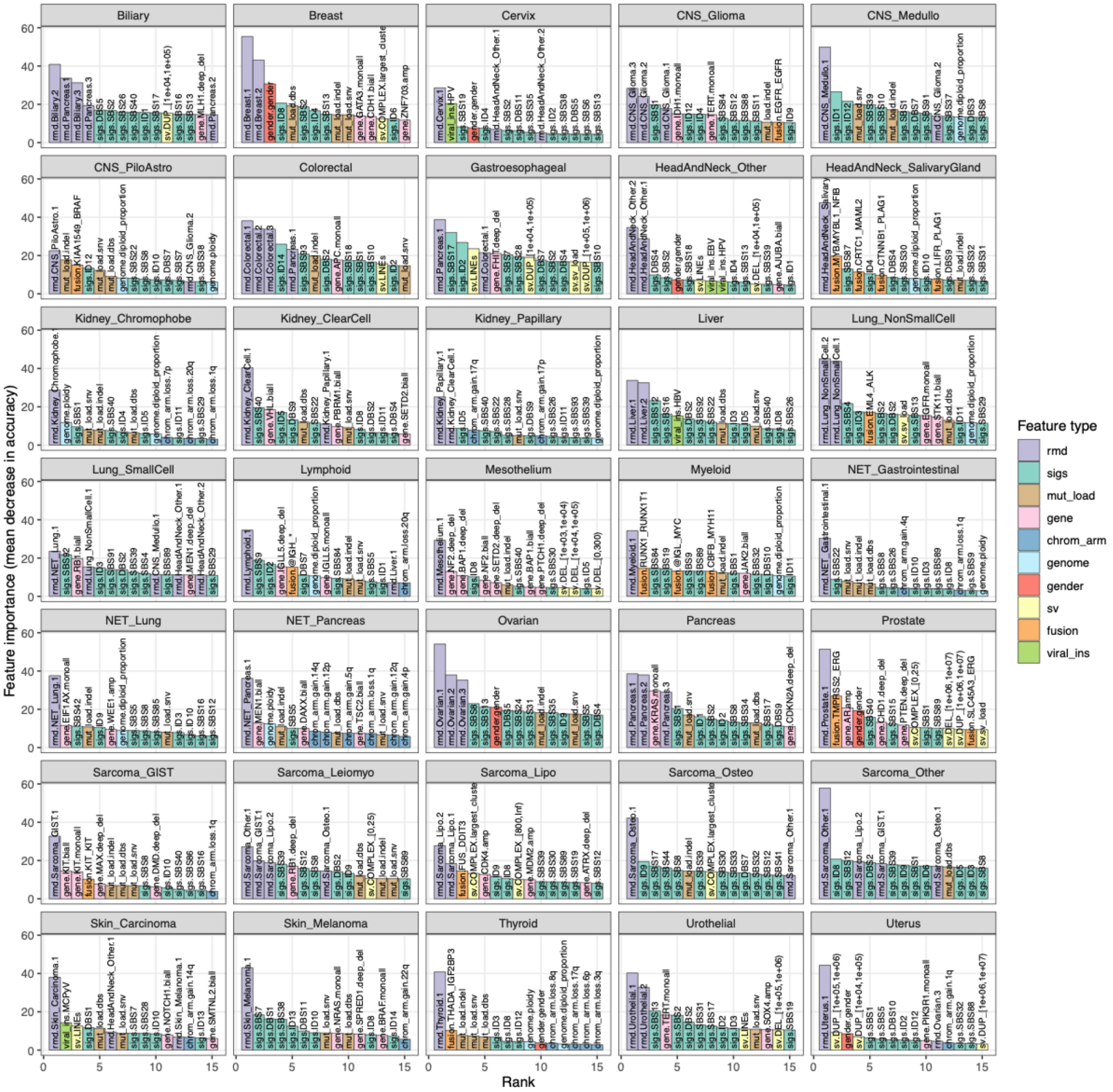

Supplementary figure 7: **Feature importances from the top 15 features for each random forest within CUPLR**. Feature importance is measured by mean decrease in accuracy across all trees in a random forest upon removing a particular feature. Feature names are in the form {feature type}.{feature name}. **Feature type definitions**; rmd: regional mutation density profiles; sigs: mutational signatures; mut_load: total number of single base substitutions, double base substitutions or indels; gene: presence of gene gain or loss of function events; chrom_arm: chromosome arm copy number fold change versus overall genome ploidy; genome: genome properties including genome ploidy, diploid proportion, whole genome duplication status; gender: sample gender as determined by copy number data; sv: structural variants; fusion: presence of gene fusions; viral_ins: presence of viral sequence insertions. See **Supplementary data 3** for the descriptions as well as feature importance values for each feature.

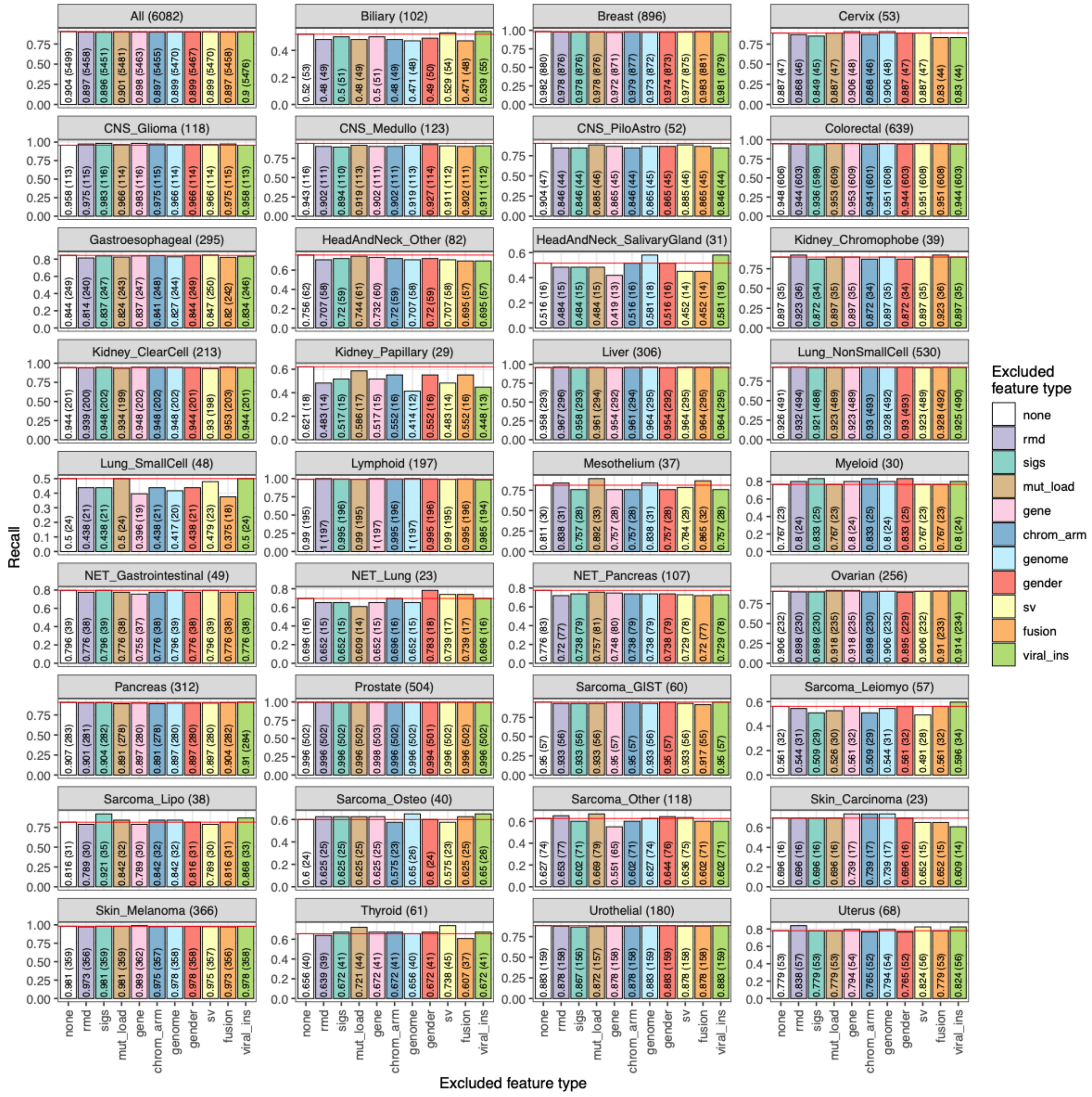

Supplementary figure 8: Cancer type prediction recall when excluding feature types from the training data. Recall (i.e. fraction of samples correctly predicted) was determined using cross validation. Panel titles indicate the cancer type and the total number of samples for that cancer type. Bars are labelled with the recall as well as the number of correctly predicted samples. The red line shows the recall when no feature types are excluded. **Feature type definitions**; rmd: regional mutation density profiles; sigs: mutational signatures; mut_load: total number of single base substitutions, double base substitutions or indels; gene: presence of gene gain or loss of function events; chrom_arm: chromosome arm copy number fold change versus overall genome ploidy; genome: genome properties including genome ploidy, diploid proportion, whole genome duplication status; gender: sample gender as determined by copy number data; sv: structural variants; fusion: presence of gene fusions; viral_ins: presence of viral sequence insertions. For a full description of each feature, see **Supplementary data 3**.

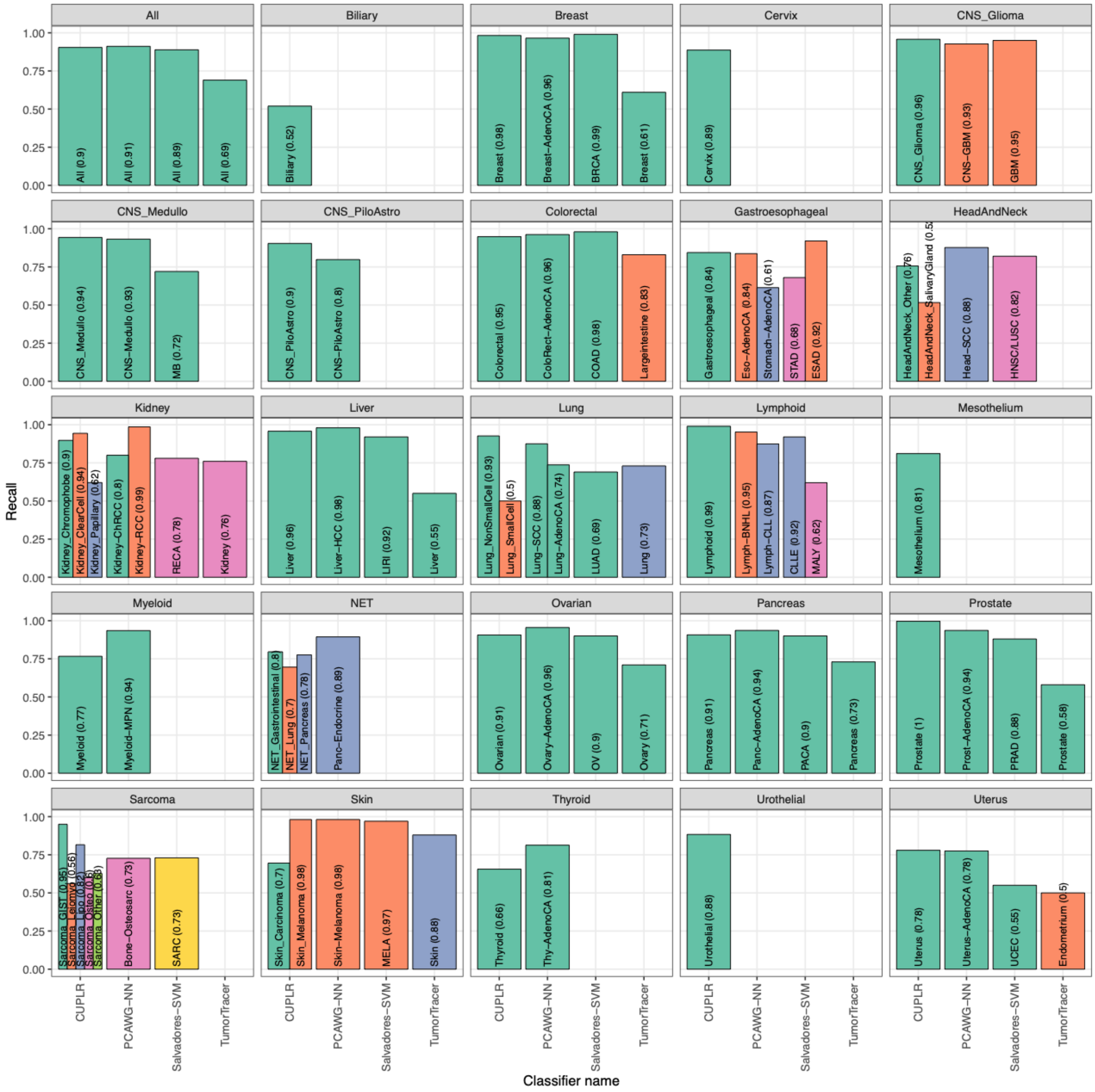

Supplementary figure 9: **Recall of CUPLR compared to other published classifiers.** Recall (i.e. fraction of samples correctly predicted) for CUPLR was determined using cross validation. Bars are labeled with the cancer type class names from the respective classifiers as well as the recall value. Cancer type class names representing the same cancer type across different classifiers are represented by the same bar colors. The names of the published classifiers refer to the following studies; PCAWG-NN: PCAWG neural network by Jiao *et al.* 2020, Salvadores-SVM: support vector machine by Salvadores *et al.* 2019, TumorTracer: Marquard *et al.* 2015.

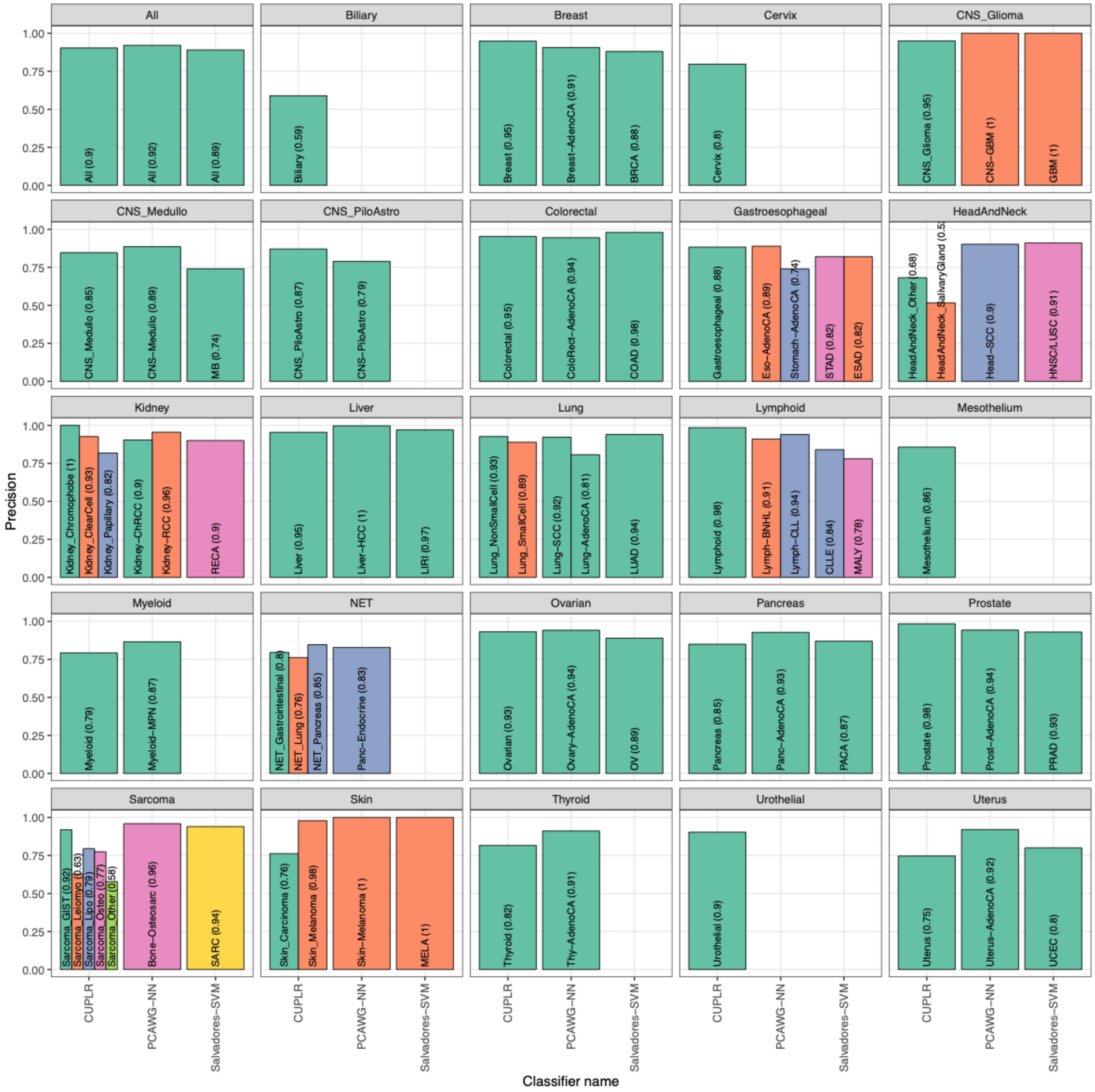

Supplementary figure 10: **Precision of CUPLR compared to other published classifiers.** Precision for CUPLR was determined using cross validation. Bars are labeled with the cancer type class names from the respective classifiers as well as the precision value. Cancer type class names representing the same cancer type across different classifiers are represented by the same bar colors. The names of the published classifiers refer to the following studies; PCAWG-NN: PCAWG neural network by Jiao *et al.* 2020, Salvadores-SVM: support vector machine by Salvadores *et al.* 2019. No precision values were provided by Marquard *et al.* 2015 for TumorTracer.

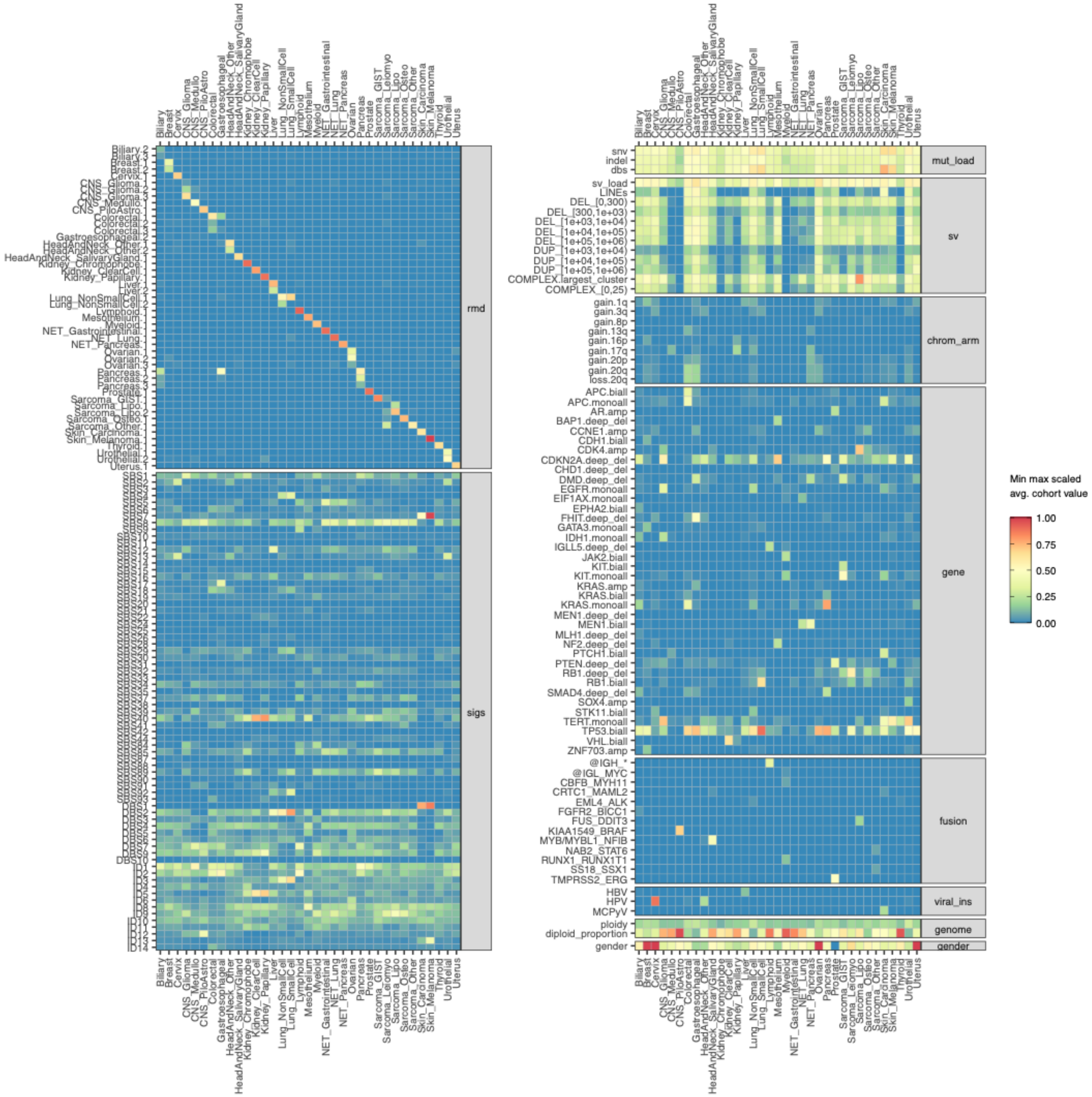

Supplementary figure 11: **Average scaled feature value per cancer type for the top 200 features**. Feature values are scaled from 0 to 1 based on the minimum and maximum value for each feature across all samples. For gender, feature values towards 1 represent more female samples and values towards 0 more male samples. **Feature type definitions**; rmd: regional mutation density profiles; sigs: mutational signatures; mut_load: total number of single base substitutions, double base substitutions or indels; gene: presence of gene gain or loss of function events; chrom_arm: chromosome arm copy number fold change versus overall genome ploidy; genome: genome properties including genome ploidy, diploid proportion, whole genome duplication status; gender: sample gender as determined by copy number data; sv: structural variants; fusion: presence of gene fusions; viral_ins: presence of viral sequence insertions. For a full description of each feature, see **Supplementary data 3**.

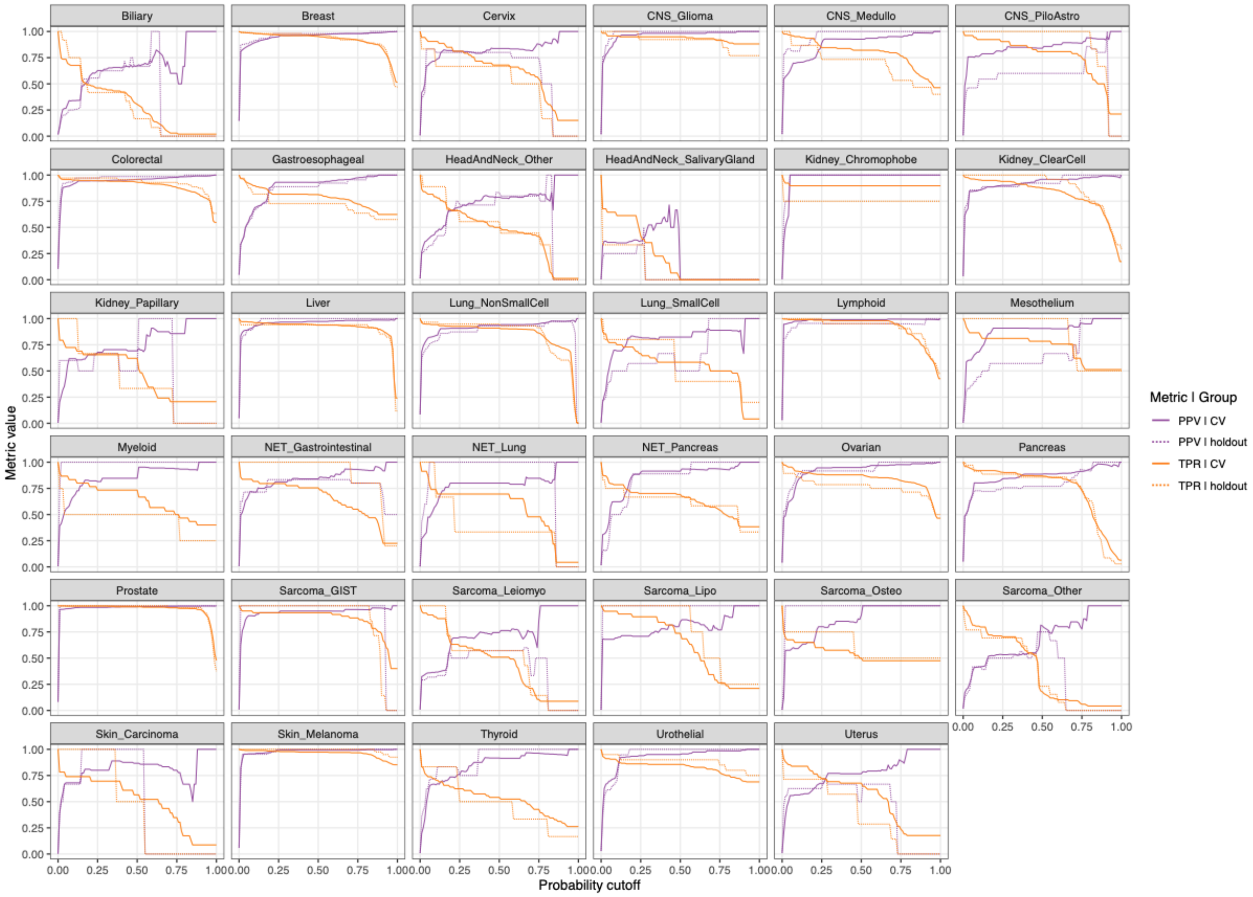

Supplementary figure 12: Positive predictive value (PPV; also known as precision) and true positive rate (TPR; also known as sensitivity or recall) curves for each binary random forest classifier within CUPLR for the based on cross-validation (CV) and holdout set predictions.

Supplementary notes

Supplementary Note 1*: Impact of confounding factors on performance*

Since the RMD profiles and mutational signatures were the most important feature types for CUPLR, we assessed whether the presence of certain confounding factors to these feature types would lead to more incorrect predictions. Firstly, this included DNA repair deficiencies including microsatellite instability (MSI) and homologous recombination deficiency (HRD) which lead to SBS/indel accumulation across the genome and could lead to different RMD profiles than would be expected for a particular cancer type. Secondly, the impact of common chemotherapies including platinum and 5-fluorouracil (5FU) was assessed as treatment induced mutations could also lead to altered RMD profiles. Furthermore, treatment associated mutational signatures could be erroneously predictive of certain cancer types (e.g. platinum signature SBS35 for ovarian cancer) despite them not being intrinsic properties of those cancer types. Lastly, smoking history was assessed for lung cancer patients to determine whether lack of smoking, and by extension the absence of SBS4 (smoking mutational signature), would reduce performance for lung cancer. We found that MSI in Gastroesophageal, Breast, and CNS_Glioma as well as HRD in Pancreas did lead to significantly more incorrect predictions ($p<0.01$, one-sided Fisher's exact test), though the number of incorrectly predicted samples was low (6, 3, 3, and 10 respectively). Overall, the presence of the majority of confounding factors did not lead to more incorrect predictions ($p\geq0.01$, one-sided Fisher's exact test; **Supplementary table 1**).

				Variable TRUE				Variable FALSE				
Variable	Class	Total	Total with variable data	Incorrect	Correct	Total	%Incorrect	Incorrect	Correct	Total	%Incorrect	p-value
has_msi	Gastroesophageal	328	328	6	1	7	85.7	48	273	321	15.0	0.00010
has_msi	Breast	996	996	3	5	8	37.5	15	973	988	1.5	0.00026
has_msi	CNS_Glioma	131	131	3	3	6	50.0	2	123	125	1.6	0.00053
has_msi	Lung_NonSmallCell	588	588	2	3	5	40.0	40	543	583	6.9	0.04341
has_msi	Urothelial	200	200	2	3	5	40.0	21	174	195	10.8	0.10217
has_msi	Uterus	75	75	4	8	12	33.3	14	49	63	22.2	0.31187
has_msi	Colorectal	710	710	2	40	42	4.8	33	635	668	4.9	0.62811
has_msi	Biliary	114	114	2	3	5	40.0	54	55	109	49.5	0.80694
has_msi	Prostate	560	560	0	17	17	0.0	2	541	543	0.4	1.00000
has_hrd	Pancreas	347	346	10	23	33	30.3	23	290	313	7.3	0.00032
has_hrd	Breast	996	989	6	145	151	4.0	9	829	838	1.1	0.01746
has_hrd	Gastroesophageal	328	320	2	3	5	40.0	45	270	315	14.3	0.15787
has_hrd	Colorectal	710	667	1	5	6	16.7	31	630	661	4.7	0.25631
has_hrd	NET_Pancreas	119	110	2	3	5	40.0	20	85	105	19.0	0.26075
has_hrd	Sarcoma_Leiomyo	64	64	3	2	5	60.0	25	34	59	42.4	0.38026
has_hrd	Lung_NonSmallCell	588	583	1	6	7	14.3	39	537	576	6.8	0.39360
has_hrd	Urothelial	200	195	1	12	13	7.7	20	162	182	11.0	0.78387
has_hrd	Biliary	114	109	3	5	8	37.5	51	50	101	50.5	0.85876
has_hrd	Ovarian	284	282	2	105	107	1.9	25	150	175	14.3	0.99998
has_hrd	Prostate	560	542	0	55	55	0.0	2	485	487	0.4	1.00000
has_smoked	Pancreas	347	83	3	24	27	11.1	2	54	56	3.6	0.19177
has_smoked	HeadAndNeck_Other	91	8	1	4	5	20.0	0	3	3	0.0	0.62500
has_smoked	Lung_NonSmallCell	588	226	7	143	150	4.7	6	70	76	7.9	0.89876
has_smoked	Gastroesophageal	328	83	1	57	58	1.7	3	22	25	12.0	0.99312
has_smoked	Breast	996	31	0	16	16	0.0	0	15	15	0.0	1.00000
has_smoked	Liver	340	25	0	14	14	0.0	2	9	11	18.2	1.00000
has_smoked	Lung_SmallCell	53	16	7	9	16	43.8	0	0	0	0.0	1.00000
has_smoked	Lymphoid	218	55	0	38	38	0.0	1	16	17	5.9	1.00000
treated_with_platinum	Gastroesophageal	328	188	12	69	81	14.8	11	96	107	10.3	0.23647
treated_with_platinum	Breast	996	605	2	61	63	3.2	7	535	542	1.3	0.23896
treated_with_platinum	Lung_SmallCell	53	22	15	6	21	71.4	0	1	1	0.0	0.31818
treated_with_platinum	HeadAndNeck_Other	91	25	6	12	18	33.3	1	6	7	14.3	0.33653
treated_with_platinum	Pancreas	347	51	3	9	12	25.0	6	33	39	15.4	0.35400
treated_with_platinum	Biliary	114	23	7	11	18	38.9	1	4	5	20.0	0.41377
treated_with_platinum	Ovarian	284	114	15	93	108	13.9	0	6	6	0.0	0.42016
treated_with_platinum	Lung_NonSmallCell	588	214	13	144	157	8.3	4	53	57	7.0	0.50910
treated_with_platinum	Uterus	75	17	6	10	16	37.5	0	1	1	0.0	0.64706
treated_with_platinum	Mesothelium	41	28	5	21	26	19.2	0	2	2	0.0	0.66931
treated_with_platinum	Urothelial	200	101	8	82	90	8.9	1	10	11	9.1	0.74650
treated_with_platinum	Colorectal	710	329	7	261	268	2.6	8	53	61	13.1	0.99970
treated_with_platinum	Sarcoma_Osteo	44	6	2	3	5	40.0	1	0	1	100.0	1.00000
treated_with_platinum	Prostate	560	478	0	9	9	0.0	2	467	469	0.4	1.00000
treated_with_platinum	Cervix	59	23	3	20	23	13.0	0	0	0	0.0	1.00000
treated_with_5FU	HeadAndNeck_Other	91	25	3	3	6	50.0	4	15	19	21.1	0.19368
treated_with_5FU	Pancreas	347	51	3	10	13	23.1	6	32	38	15.8	0.41389
treated_with_5FU	Breast	996	605	3	165	168	1.8	6	431	437	1.4	0.47703
treated_with_5FU	Gastroesophageal	328	188	1	4	5	20.0	22	161	183	12.0	0.48316
treated_with_5FU	Biliary	114	23	1	4	5	20.0	7	11	18	38.9	0.91076
treated_with_5FU	Colorectal	710	329	2	80	82	2.4	13	234	247	5.3	0.92366

Supplementary table 1: Comparison of the number of correctly and incorrectly predicted samples between patients with and without: microsatellite instability (MSI), homologous recombination deficiency (HRD), smoking history, treatment with platinum, and treatment with 5-fluorouracil (5FU). One-sided Fisher's exact tests were performed to determine if there were more incorrectly predicted patients with versus without the respective variable. Only cancer types with at least 5 patients in total being positive for the respective variable (i.e. Variable TRUE: Total >= 5) are shown.

As subclonal variants are enriched for treatment induced mutations, we also compared the performance of CUPLR when the model was trained on RMD and mutational signatures that were extracted from all mutations versus only clonal mutations (i.e. treatment induced mutations excluded) (**Supplementary figure 13**). No differences in overall recall were found (all mutations: 90% vs. clonal mutations 89%), with the exception of decreased recall when only clonal mutations were used in 5 cancer types: CNS_PiloAstro (90% vs 77%), Kidney_Chromophobe (90% vs. 72%), Myeloid (77% vs. 63%), NET_Gastrointestinal (80% vs. 47%) and Sarcoma_GIST (95% vs 82%). Given the similar performance between training on all mutations versus only clonal mutations, treatment induced mutations likely have minimal impact on the performance of CUPLR for the majority of cancer types.

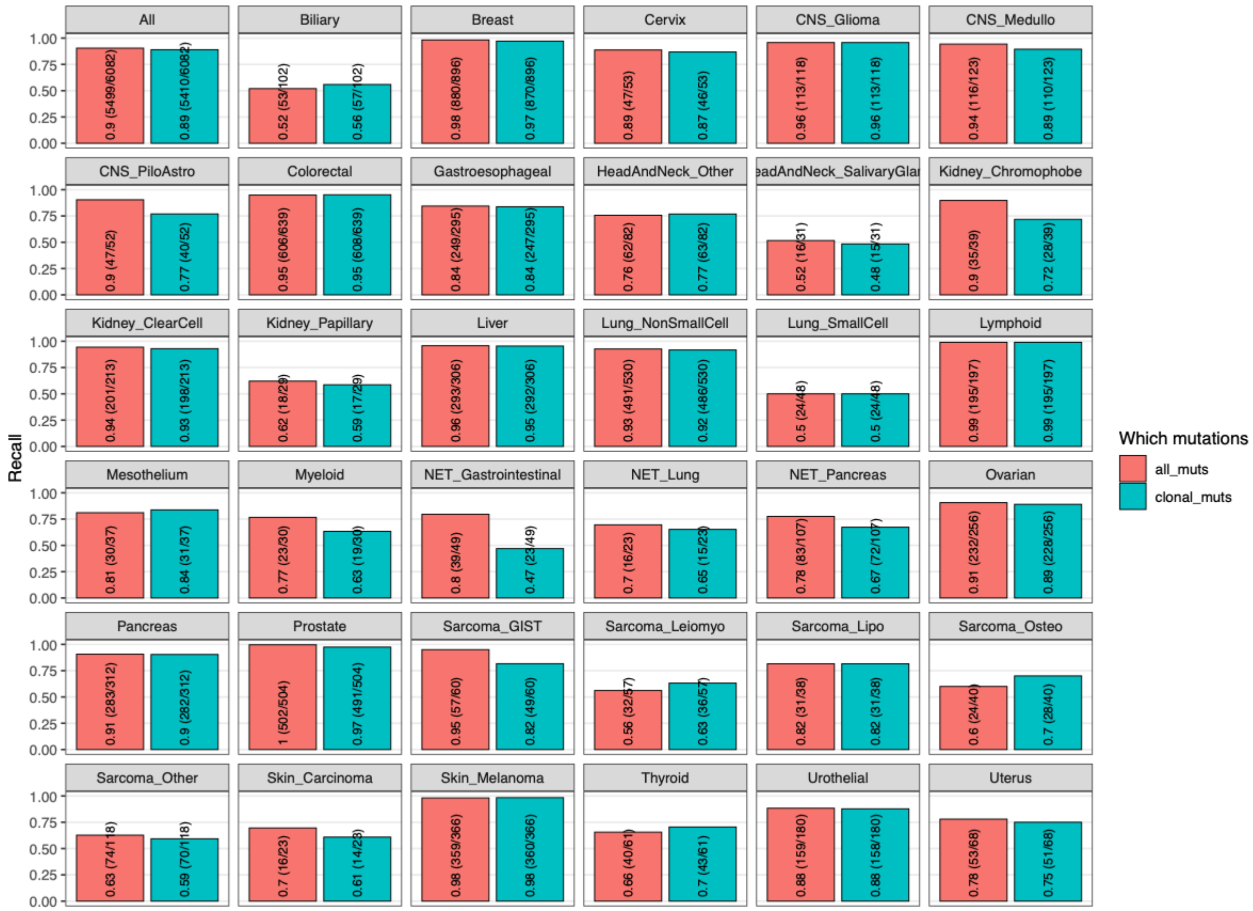

Supplementary figure 13: Comparison of cancer type prediction recall when using all mutations versus only clonal mutations to generate the regional mutational density and mutational signature features. Recall (i.e. fraction of samples correctly predicted) was determined using cross validation. Bars are labeled with the recall value, as well as in brackets the number of correctly predicted samples and the total number of samples.

Supplementary Note 2: Impact of sequencing coverage on performance

Our dataset consisted of patients with tumor samples sequenced at roughly 30x, 60x and 90x coverage (with normal samples all sequenced at ~30x coverage). Thus, to assess the impact of sequencing depth, we compared the performance (based on cross-validation predictions) between these coverages for all samples, as well as for cancer types with at least 3 samples at each coverage (20 cancer types; **Supplementary figure 14**).

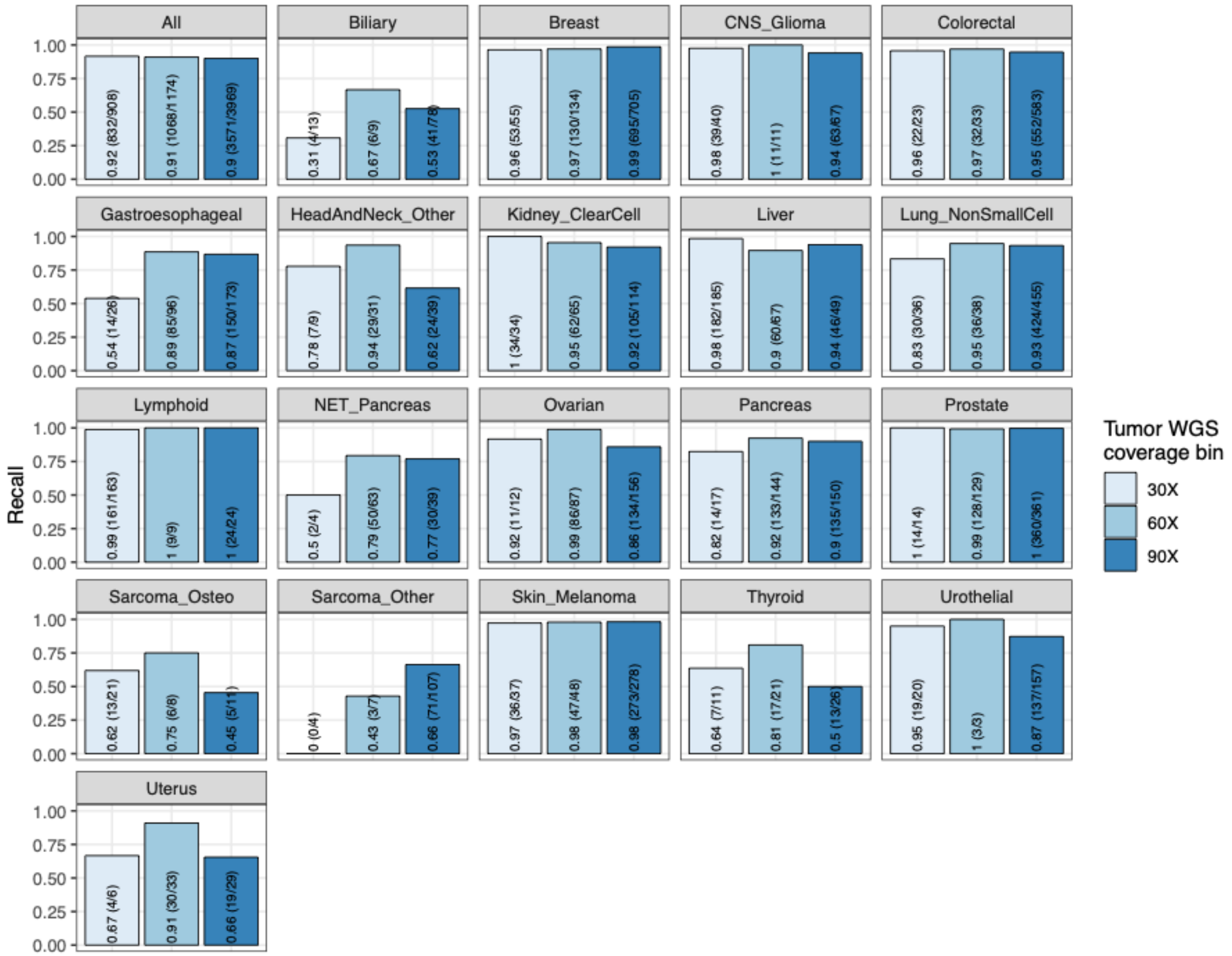

Supplementary figure 14: Comparison of performance between samples sequenced at different coverages. Recall (i.e. fraction of samples correctly predicted) was determined by testing on the opposite dataset. Bars are labeled with the recall value, as well as in brackets the number of correctly predicted samples and the total number of samples.

Overall, we found similar performance across difference sequencing depths (30x: 92%, 60x: 91%, 90x: 90%). Per cancer type, 12 cancer types had comparable recall across the 3 coverages, including Breast, CNS_Glioma, Colorectal, Kidney_ClearCell, Liver, Lung_NonSmallCell, Lymphoid, Ovarian, Pancreas, Prostate, Skin_Melanoma, Urothelial. For 4 cancer types (HeadAndNeck_Other, Sarcoma_Osteo, Thyroid and Uterus) there was no apparent correlation between coverage and recall. Lastly, recall was lower at 30x coverage for Biliary, Gastroesophageal, NET_Pancreas, with Sarcoma_Other also having lower recall at 30x and 60x.

In summary, 30x coverage should be sufficient for reliable CUPLR predictions for most cancer types, but >=60x coverage would lead to the most reliable predictions.

Supplementary Note 3: Impact of batch effects

To assess the potential for CUPLR to be overfit on the Hartwig and/or PCAWG datasets (i.e. the potential presence of batch effects), we trained a model solely on Hartwig samples (Hartwig-only) and another model solely on PCAWG samples (PCAWG-only), and determined performance by testing on the opposite dataset (**Supplementary figure 15**). Hartwig-only and PCAWG-only models were trained with equal numbers of samples to avoid better performance solely due to higher sample size.

Overall, we found that both models achieved similar recall (Hartwig-only: 90%, PCAWG-only: 86%). For the 21 cancer types with sufficient sample sizes for training (>=15 samples in both Hartwig and PCAWG cohorts), 13 of these also had comparable recall between the two models (+/-10%). These data indicate that batch effects for most cancer types are minimal.

However, for 6 cancer types (Cervix, Colorectal, HeadAndNeck_Other, Ovarian, Sarcoma_Lipo, Thyroid), recall of the Hartwig-only model was much higher than of the PCAWG-only model. Conversely, the PCAWG-only model had higher recall than the Hartwig-only model for 2 cancer types (Biliary, Sarcoma_Leiomyo). These results indeed indicate potential technical and/or biological differences between the Hartwig and PCAWG cohorts for these cancer types. These results nevertheless highlight the importance of incorporating both Hartwig and PCAWG samples for training (e.g. to mitigate the impact from treatment associated mutations which may be more abundant in Hartwig samples).

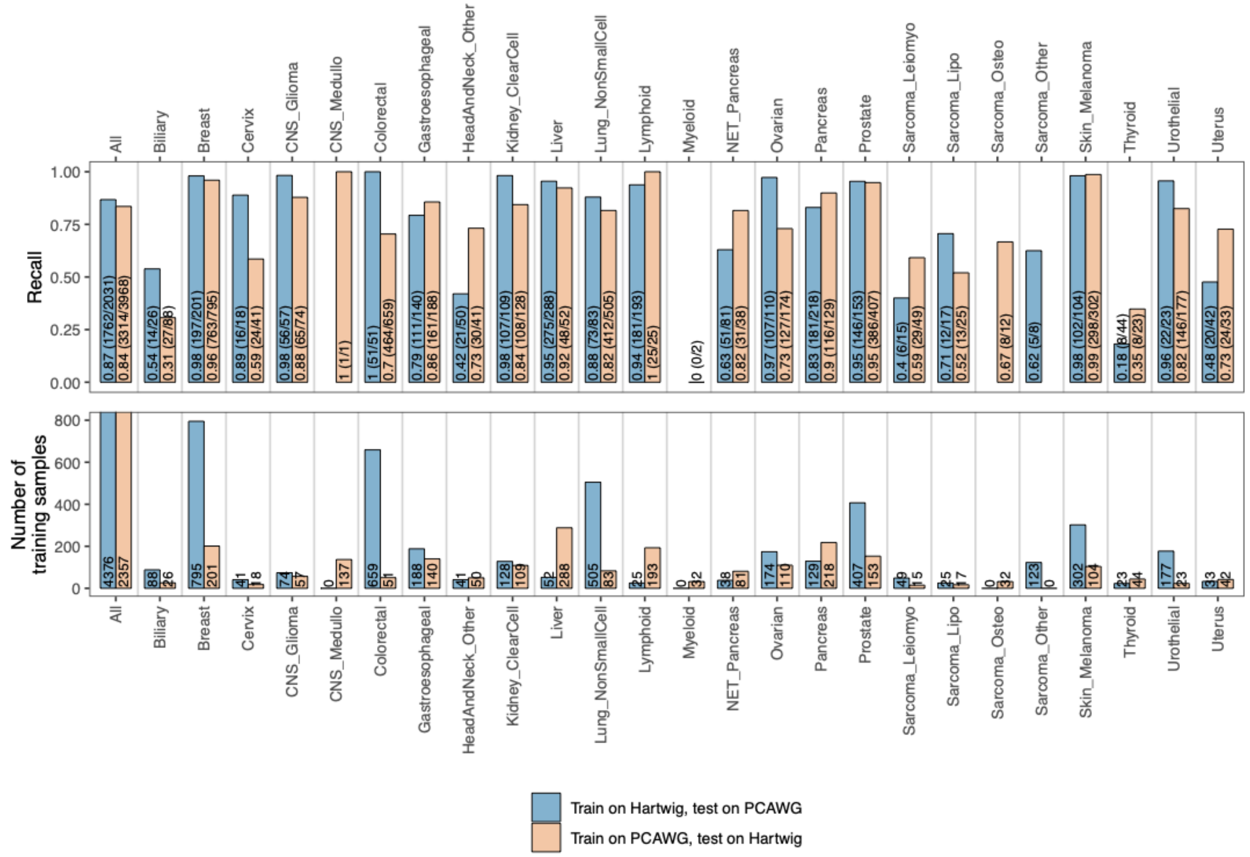

Supplementary figure 15: Comparison of performance between a CUP classifier model trained solely on Hartwig samples (Hartwig-only) and another model solely on PCAWG samples (PCAWG-only). Only cancer types with at least 15 samples in both Hartwig and PCAWG cohorts were included for training. Recall (i.e. fraction of samples correctly predicted) was determined by testing on the opposite dataset. Bars are labeled with the recall value, as well as in brackets the number of correctly predicted samples and the total number of samples.
