# Supplementary material for: Machine learning-based tissue of origin classification for cancer of unknown primary diagnostics using genome-wide mutation features
Source: Nat Commun. 2022 Jul 11;13:4013. doi: 10.1038/s41467-022-31666-w (PMC9273599; doi:10.1038/s41467-022-31666-w)
Supplement: Supplementary file 3 — Reporting Summary [file 41467_2022_31666_MOESM3_ESM.pdf]

nature portfolio

Corresponding author(s): Edwin Cuppen

Last updated by author(s): Jun 10, 2022

# Reporting Summary

Nature Portfolio wishes to improve the reproducibility of the work that we publish. This form provides structure for consistency and transparency in reporting. For further information on Nature Portfolio policies, see our Editorial Policies and the Editorial Policy Checklist.

## Statistics

For all statistical analyses, confirm that the following items are present in the figure legend, table legend, main text, or Methods section.

| n/a | Confirmed | |
|---|---|---|
| ☐ | ☒ | The exact sample size (*n*) for each experimental group/condition, given as a discrete number and unit of measurement |
| ☐ | ☒ | A statement on whether measurements were taken from distinct samples or whether the same sample was measured repeatedly |
| ☐ | ☒ | The statistical test(s) used AND whether they are one- or two-sided<br>*Only common tests should be described solely by name; describe more complex techniques in the Methods section.* |
| ☒ | ☐ | A description of all covariates tested |
| ☐ | ☒ | A description of any assumptions or corrections, such as tests of normality and adjustment for multiple comparisons |
| ☐ | ☒ | A full description of the statistical parameters including central tendency (e.g. means) or other basic estimates (e.g. regression coefficient) AND variation (e.g. standard deviation) or associated estimates of uncertainty (e.g. confidence intervals) |
| ☐ | ☒ | For null hypothesis testing, the test statistic (e.g. *F*, *t*, *r*) with confidence intervals, effect sizes, degrees of freedom and *P* value noted<br>*Give P values as exact values whenever suitable.* |
| ☒ | ☐ | For Bayesian analysis, information on the choice of priors and Markov chain Monte Carlo settings |
| ☒ | ☐ | For hierarchical and complex designs, identification of the appropriate level for tests and full reporting of outcomes |
| ☐ | ☒ | Estimates of effect sizes (e.g. Cohen's *d*, Pearson's *r*), indicating how they were calculated |

*Our web collection on statistics for biologists contains articles on many of the points above.*

## Software and code

Policy information about availability of computer code

Data collection

No software was used for data collection

Data analysis

Somatic mutation data of the CPCT, DRUP and WIDE projects were kindly shared by Hartwig on 6 February 2020 with an update received on 20 October 2021.

The PCAWG samples were reanalyzed with the Hartwig somatic variant calling pipeline (https://github.com/hartwigmedical/pipeline5) which was hosted on the Google Cloud Platform using Platinum (https://github.com/hartwigmedical/platinum). This pipeline uses the following software packages:
BWA (v0.7.17): read mapping
GATK (v3.8.0) Haplotype Caller: calling germline variants in the reference sample
SAGE (v2.2): somatic SMNVs and indels calling
GRIDSS (v2.9.3): simple and complex structural variant calling
PURPLE (v2.53): combines B-allele frequency (BAF) from AMBER (v3.3), read depth ratios from COBALT (v1.7), and structural variants from GRIDSS to estimate copy number profiles, variant allele frequency (VAF) and variant clonality. PURPLE also determines sample gender based on sex chromosome ploidy.
LINX (v1.17): interpretation of simple mutations and structural variants

All code used for developing CUPLR can be found at https://github.com/UMCUGenetics/cuplr. This repo is composed of 4 subpackages:
https://github.com/UMCUGenetics/cuplr/tree/master/cuplr (training and running CUPLR)
https://github.com/UMCUGenetics/cuplr/tree/master/featureExtractor (extraction of input features for CUPLR)
https://github.com/UMCUGenetics/cuplr/tree/master/statsExtra (statistics for feature selection during training)
https://github.com/UMCUGenetics/cuplr/tree/master/nmf (non-negative matrix factorization for generating regional mutational density

profiles)

Other supporting code for CUPLR can be found at the following github repos:
https://github.com/UMCUGenetics/mutSigExtractor/ (extraction of mutational signatures)
https://github.com/UMCUGenetics/mltoolkit (classification statistics)

For manuscripts utilizing custom algorithms or software that are central to the research but not yet described in published literature, software must be made available to editors and reviewers. We strongly encourage code deposition in a community repository (e.g. GitHub). See the Nature Portfolio guidelines for submitting code & software for further information.

## Data

Policy information about availability of data

All manuscripts must include a data availability statement. This statement should provide the following information, where applicable:

- Accession codes, unique identifiers, or web links for publicly available datasets
- A description of any restrictions on data availability
- For clinical datasets or third party data, please ensure that the statement adheres to our policy

Metastatic WGS data and corresponding metadata have been obtained from the Hartwig Medical Foundation and provided under data request numbers DR-104. Both WGS data and metadata is freely available for academic use from the Hartwig Medical Foundation through standardized procedures and request forms can be found at https://www.hartwigmedicalfoundation.nl. For access to identifying data (e.g. germline or raw read data) for the PCAWG cohort, researchers will need to request access via the ICGC Data Access Compliance Office (DACO; https://daco.icgc.org/).

The extracted features for each sample and used to develop CUPLR is available at https://doi.org/10.5281/zenodo.5939805. All code used for data processing and generating figures is available at https://github.com/UMCUGenetics/CUPLR.

# Field-specific reporting

Please select the one below that is the best fit for your research. If you are not sure, read the appropriate sections before making your selection.

- [x] Life sciences
- [ ] Behavioural & social sciences
- [ ] Ecological, evolutionary & environmental sciences

For a reference copy of the document with all sections, see nature.com/documents/nr-reporting-summary-flat.pdf

# Life sciences study design

All studies must disclose on these points even when the disclosure is negative.

| | |
|---|---|
| Sample size | We requested the data for all possible samples from the Hartwig and PCAWG cohorts. The Hartwig cohort included 4902 metastatic tumor samples from 4572 patients. The PCAWG cohort consisted of 2835 tumor samples from unique patients. |
| Data exclusions | For the Hartwig cohort, only a single sample of each patient was used for this study. To do this, we selected the tumor sample with the earliest biopsy date, and if this information did not exist we selected the sample with the highest tumor purity. However, some Hartwig patients had biopsies from different primary tumor locations. In these cases, we kept at least one sample from each primary tumor location, and when there were multiple samples from the same primary tumor location, we applied the aforementioned biopsy date and tumor purity filtering criteria.<br><br>For the PCAWG cohort, samples with <0.2 tumor purity were excluded from this study as somatic variant calling was not reliable for these samples. PCAWG samples that were gray- or blacklisted by the PCAWG consortium were also excluded (https://dcc.icgc.org/releases/PCAWG/donors_and_biospecimens).<br><br>For both cohorts, we only kept samples with >=50 SNVs/indels, and removed an additional set of samples for several reasons including due to failed variant calling, insufficient informed consent for use of the WGS data, and one duplicate PCAWG sample (DO217844) that was already included in the Hartwig cohort. Lastly, we only selected samples from cancer types with at least 15 samples. Ultimately, we selected 4391 Hartwig samples and 2365 PCAWG samples for training, as well as 141 Hartwig CUP samples for the CUP analysis. |
| Replication | We used both cross-validation of the training set and prediction on a holdout test set to assess the performance of CUPLR |
| Randomization | All samples were randomly assigned to the training set and test set. Training set samples were also randomly assigned to different group when performing cross-validation |
| Blinding | For evaluation of model performance, cancer type labels were blinded when making predictions on the test set as well as during cross-validation |

# Reporting for specific materials, systems and methods

We require information from authors about some types of materials, experimental systems and methods used in many studies. Here, indicate whether each material, system or method listed is relevant to your study. If you are not sure if a list item applies to your research, read the appropriate section before selecting a response.

## Materials & experimental systems

| n/a | Involved in the study |
|---|---|
| ☒ | ☐ Antibodies |
| ☒ | ☐ Eukaryotic cell lines |
| ☒ | ☐ Palaeontology and archaeology |
| ☒ | ☐ Animals and other organisms |
| ☒ | ☐ Human research participants |
| ☒ | ☐ Clinical data |
| ☒ | ☐ Dual use research of concern |

## Methods

| n/a | Involved in the study |
|---|---|
| ☒ | ☐ ChIP-seq |
| ☒ | ☐ Flow cytometry |
| ☒ | ☐ MRI-based neuroimaging |
