# Supplementary material for: Machine learning-based tissue of origin classification for cancer of unknown primary diagnostics using genome-wide mutation features
Source: Nat Commun. 2022 Jul 11;13:4013. doi: 10.1038/s41467-022-31666-w (PMC9273599; doi:10.1038/s41467-022-31666-w)
Supplement: Supplementary file 9 — Supplementary Data 6 [file 41467_2022_31666_MOESM9_ESM.pdf]

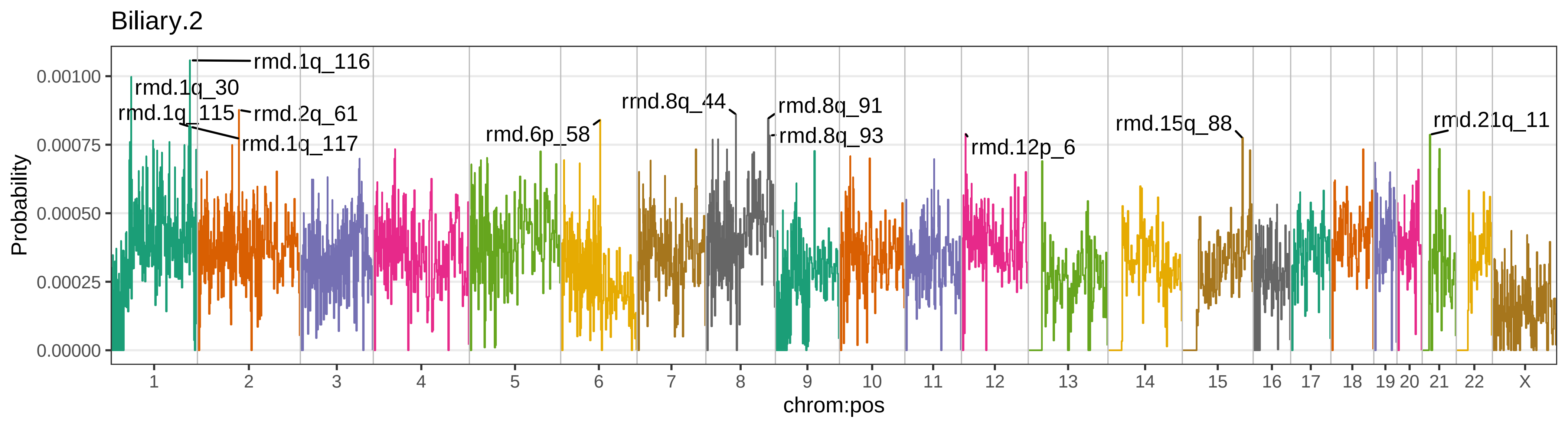
Biliary.2
Probability
0.00100
0.00075
0.00050
0.00025
0.00000
rmd.1q_116
rmd.1q_30
rmd.1q_115
rmd.2q_61
rmd.1q_117
rmd.6p_58
rmd.8q_44
rmd.8q_91
rmd.8q_93
rmd.12p_6
rmd.15q_88
rmd.21q_11
1
2
3
4
5
6
7
8
9
10
11
12
13
14
15
16
17
18
19
20
21
22
X
chrom:pos

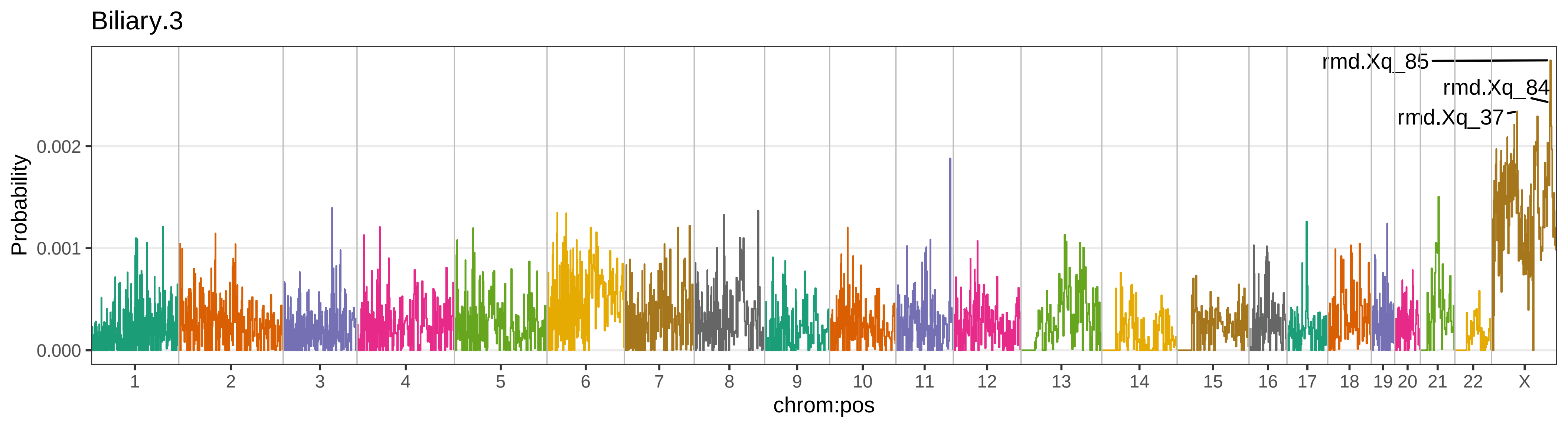
Biliary.3
Probability
0.000
0.001
0.002
chrom:pos
1
2
3
4
5
6
7
8
9
10
11
12
13
14
15
16
17
18
19
20
21
22
X
rmd.Xq_85
rmd.Xq_84
rmd.Xq_37

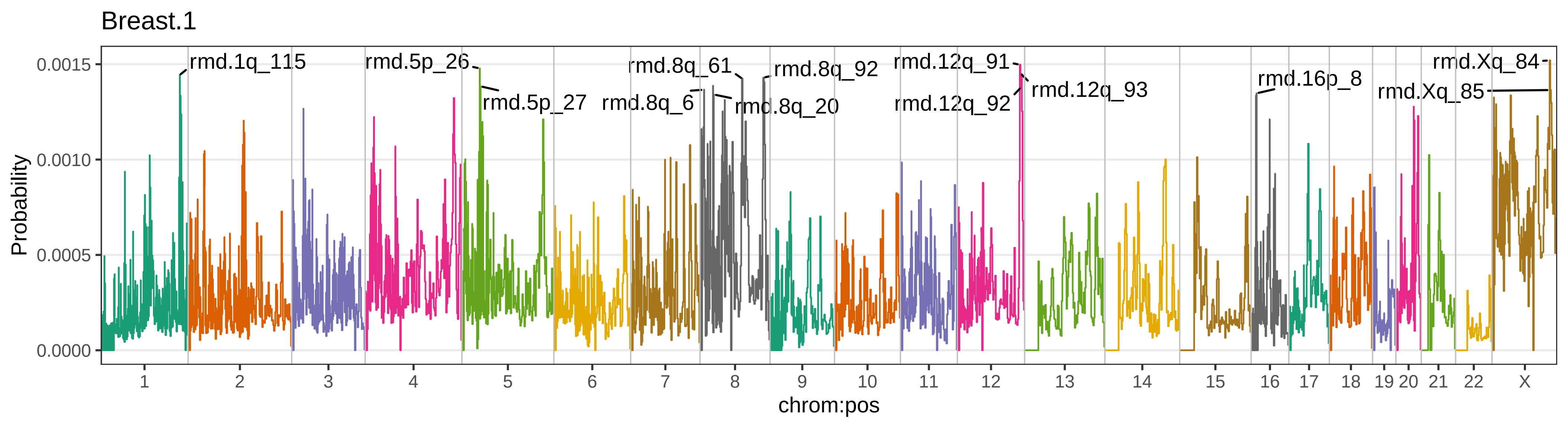
Breast.1
Probability
0.0015
0.0010
0.0005
0.0000
rmd.1q_115
rmd.5p_26
rmd.5p_27
rmd.8q_61
rmd.8q_6
rmd.8q_20
rmd.8q_92
rmd.12q_91
rmd.12q_92
rmd.12q_93
rmd.16p_8
rmd.Xq_84
rmd.Xq_85
1 2 3 4 5 6 7 8 9 10 11 12 13 14 15 16 17 18 19 20 21 22 X
chrom:pos

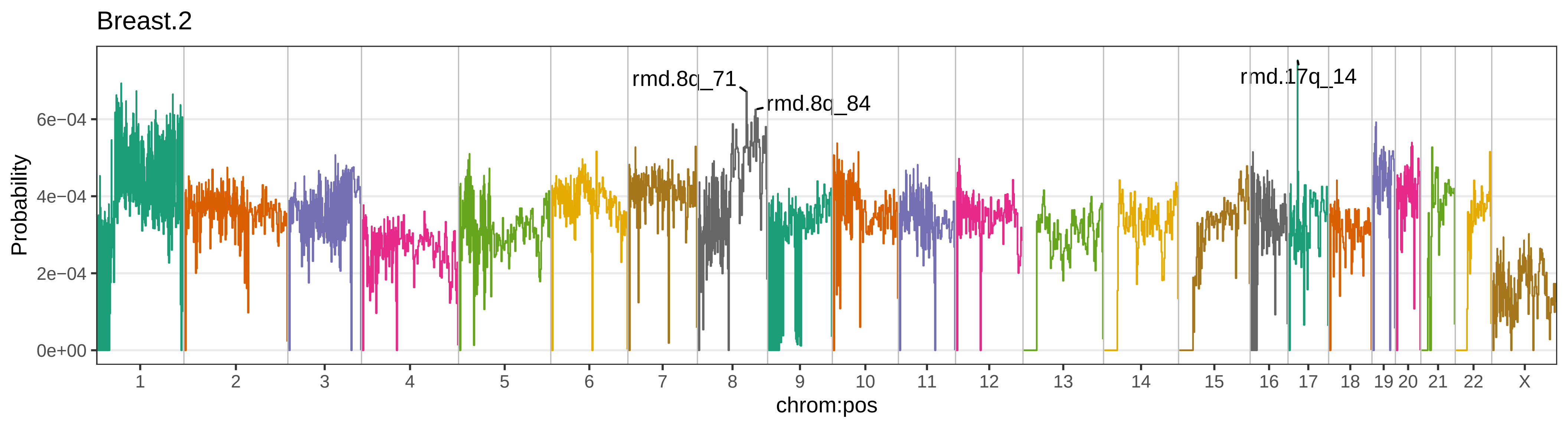
Breast.2
rmd.8q_71
rmd.8q_84
rmd.17q_14
Probability
6e−04
4e−04
2e−04
0e+00
1
2
3
4
5
6
7
8
9
10
11
12
13
14
15
16
17
18
19
20
21
22
X
chrom:pos

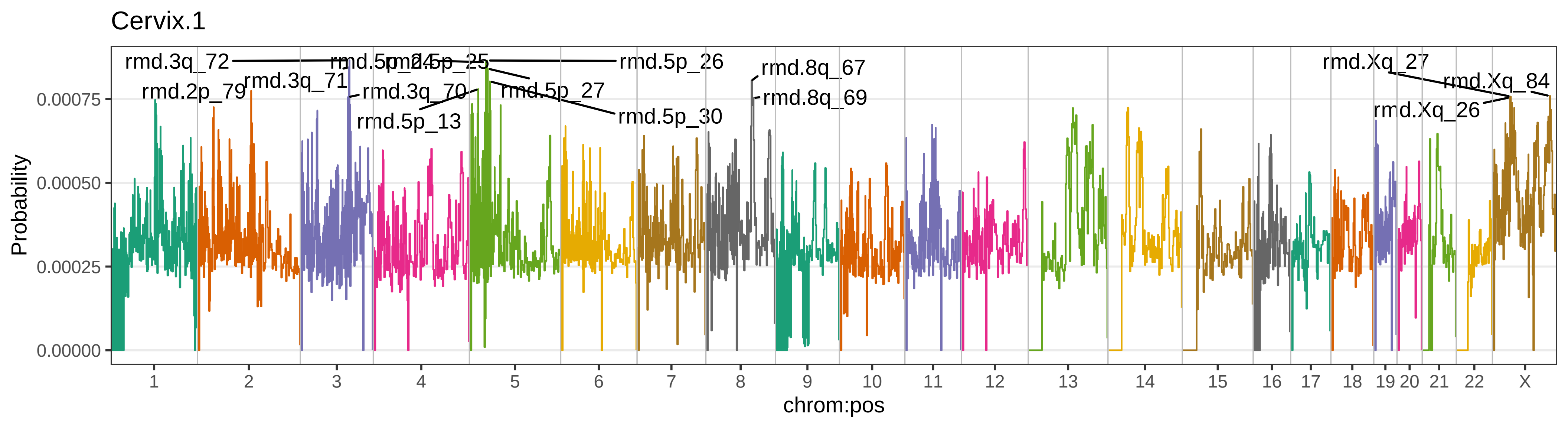
Cervix.1
rmd.3q_72
rmd.5p_24
rmd.5p_25
rmd.5p_26
rmd.8q_67
rmd.Xq_27
rmd.2p_79
rmd.3q_71
rmd.3q_70
rmd.5p_27
rmd.8q_69
rmd.Xq_84
rmd.5p_13
rmd.5p_30
rmd.Xq_26
Probability
0.00075
0.00050
0.00025
0.00000
1
2
3
4
5
6
7
8
9
10
11
12
13
14
15
16
17
18
19
20
21
22
X
chrom:pos

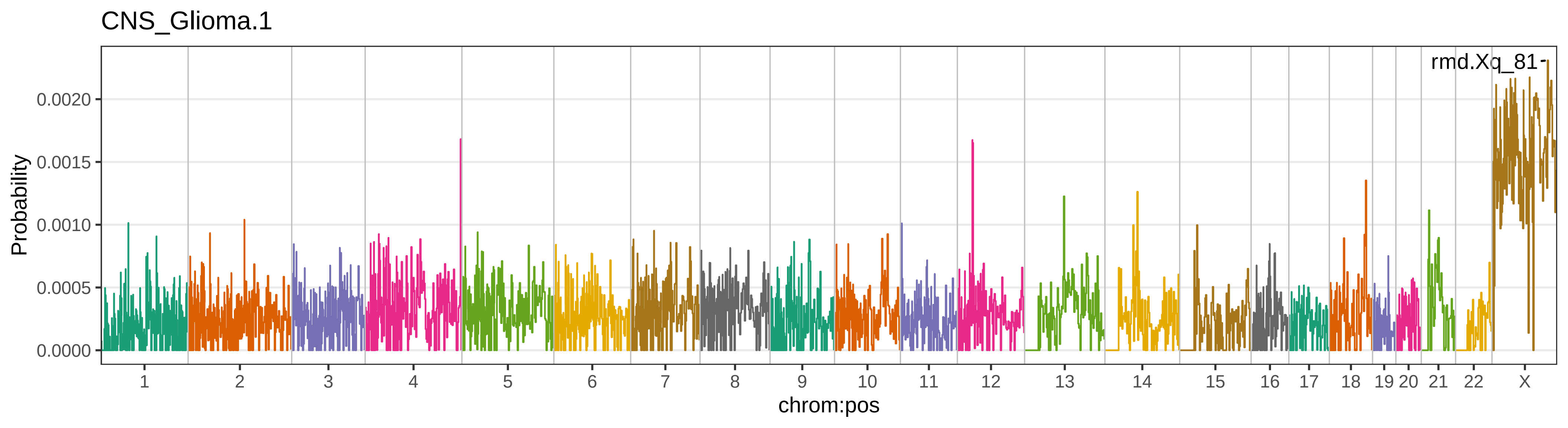
CNS_Glioma.1
rmd.Xq_81
Probability
0.0020
0.0015
0.0010
0.0005
0.0000
1
2
3
4
5
6
7
8
9
10
11
12
13
14
15
16
17
18
19
20
21
22
X
chrom:pos

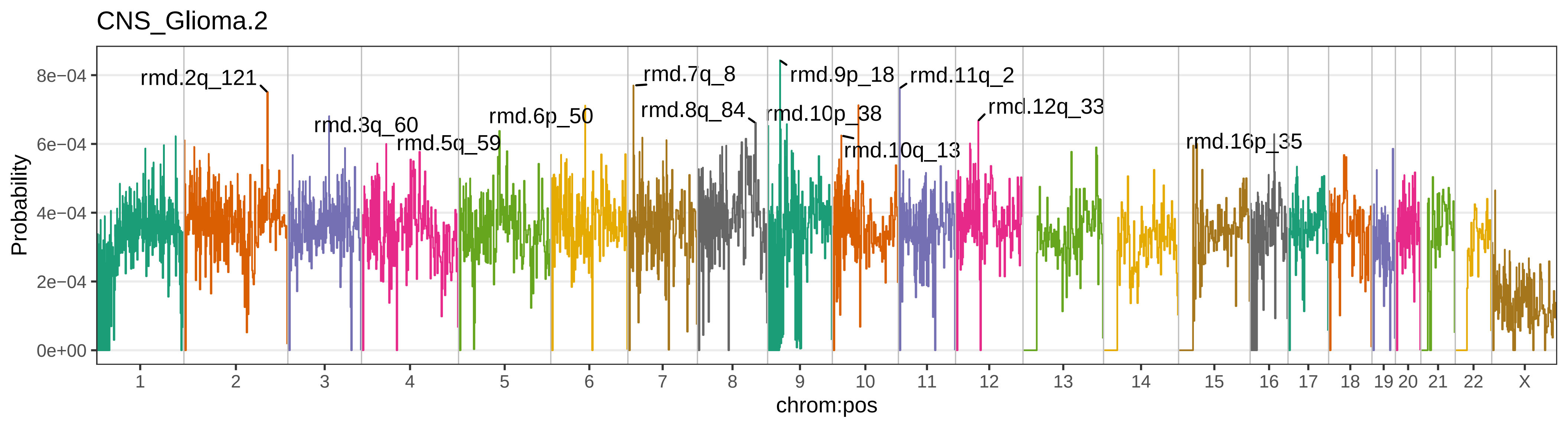
CNS_Glioma.2
Probability
8e−04
6e−04
4e−04
2e−04
0e+00
rmd.2q_121
rmd.3q_60
rmd.5q_59
rmd.6p_50
rmd.7q_8
rmd.8q_84
rmd.9p_18
rmd.10p_38
rmd.10q_13
rmd.11q_2
rmd.12q_33
rmd.16p_35
1
2
3
4
5
6
7
8
9
10
11
12
13
14
15
16
17
18
19
20
21
22
X
chrom:pos

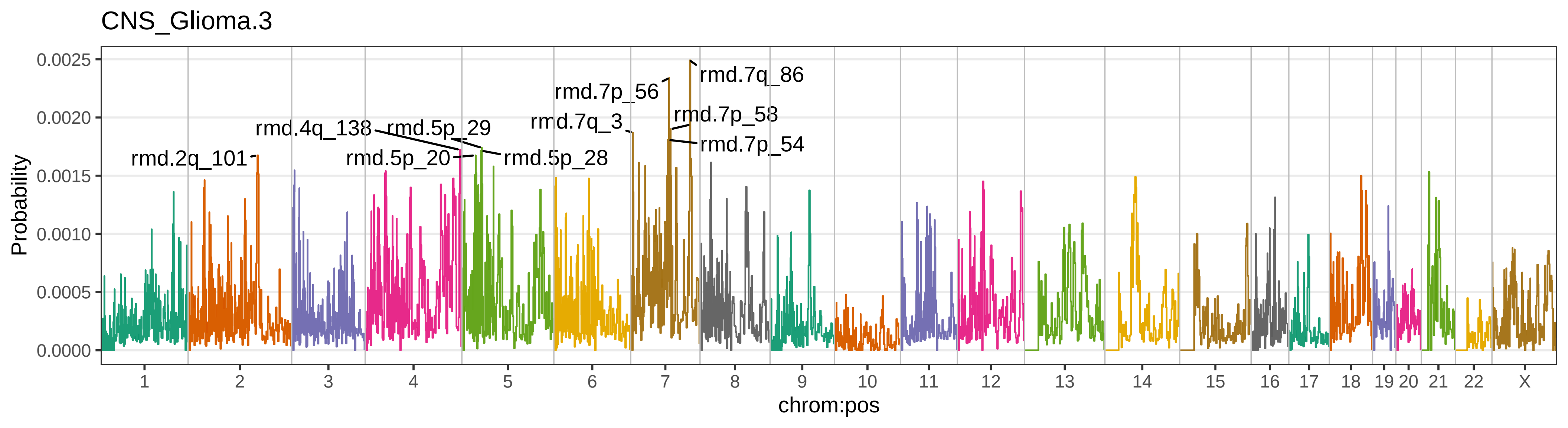

CNS_Glioma.3
Probability
chrom:pos
rmd.7q_86
rmd.7p_56
rmd.7p_58
rmd.7q_3
rmd.4q_138
rmd.5p_29
rmd.7p_54
rmd.2q_101
rmd.5p_20
rmd.5p_28
0.0025
0.0020
0.0015
0.0010
0.0005
0.0000
1 2 3 4 5 6 7 8 9 10 11 12 13 14 15 16 17 18 19 20 21 22 X

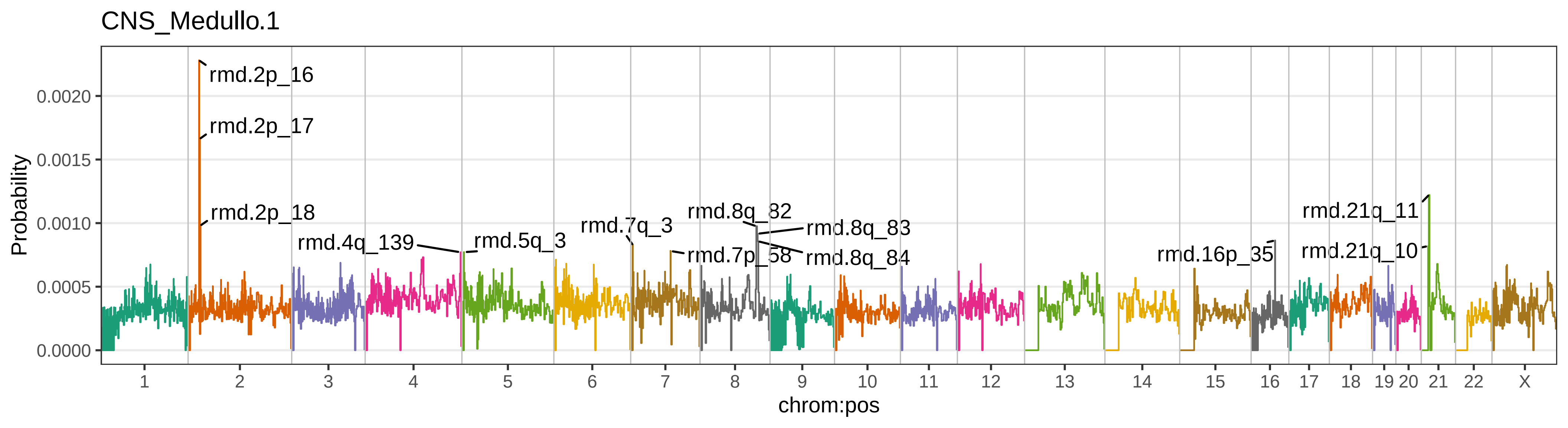
CNS_Medullo.1
Probability
0.0020
0.0015
0.0010
0.0005
0.0000
rmd.2p_16
rmd.2p_17
rmd.2p_18
rmd.4q_139
rmd.5q_3
rmd.7q_3
rmd.7p_58
rmd.8q_82
rmd.8q_83
rmd.8q_84
rmd.16p_35
rmd.21q_11
rmd.21q_10
1 2 3 4 5 6 7 8 9 10 11 12 13 14 15 16 17 18 19 20 21 22 X
chrom:pos

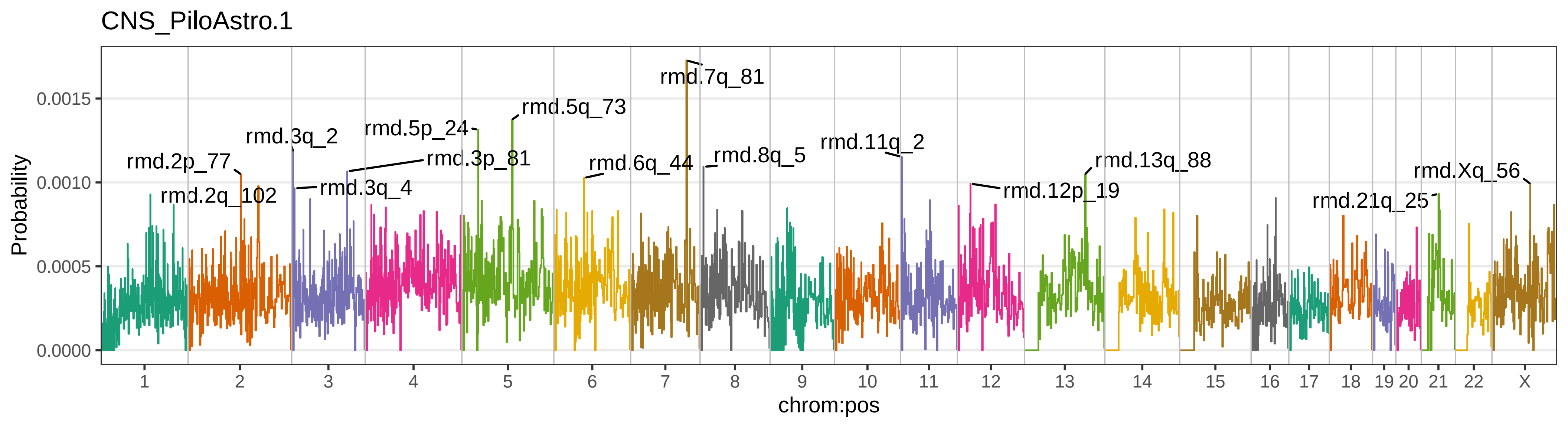

CNS_PiloAstro.1
Probability
0.0015
0.0010
0.0005
0.0000
rmd.7q_81
rmd.5q_73
rmd.5p_24
rmd.3q_2
rmd.3p_81
rmd.2p_77
rmd.6q_44
rmd.8q_5
rmd.11q_2
rmd.13q_88
rmd.Xq_56
rmd.3q_4
rmd.2q_102
rmd.12p_19
rmd.21q_25
1 2 3 4 5 6 7 8 9 10 11 12 13 14 15 16 17 18 19 20 21 22 X
chrom:pos

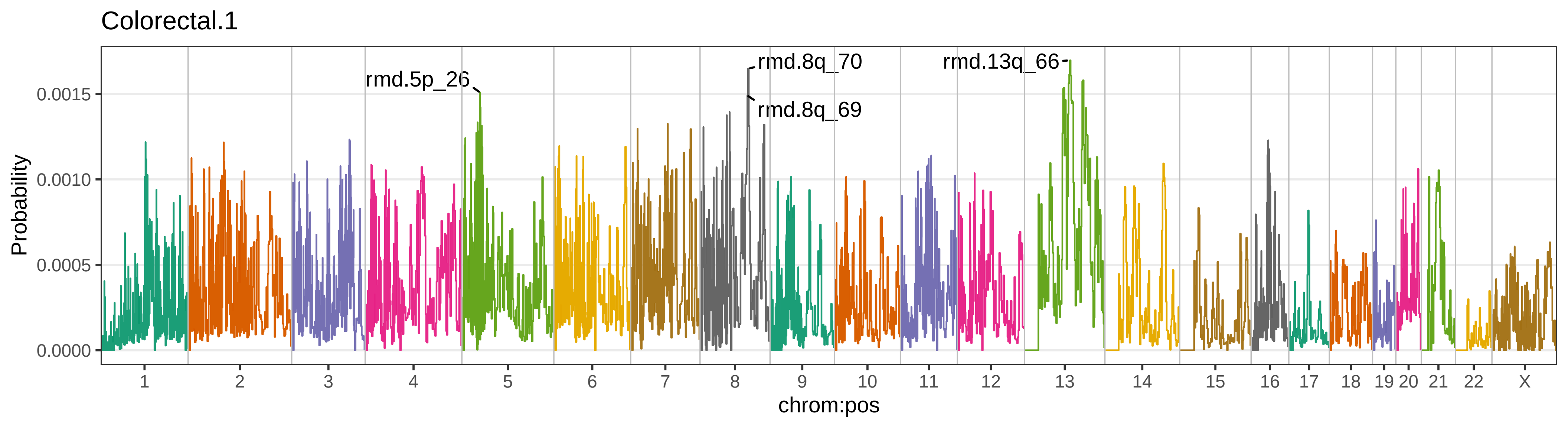
Colorectal.1
Probability
0.0015
0.0010
0.0005
0.0000
rmd.5p_26
rmd.8q_70
rmd.8q_69
rmd.13q_66
1
2
3
4
5
6
7
8
9
10
11
12
13
14
15
16
17
18
19
20
21
22
X
chrom:pos

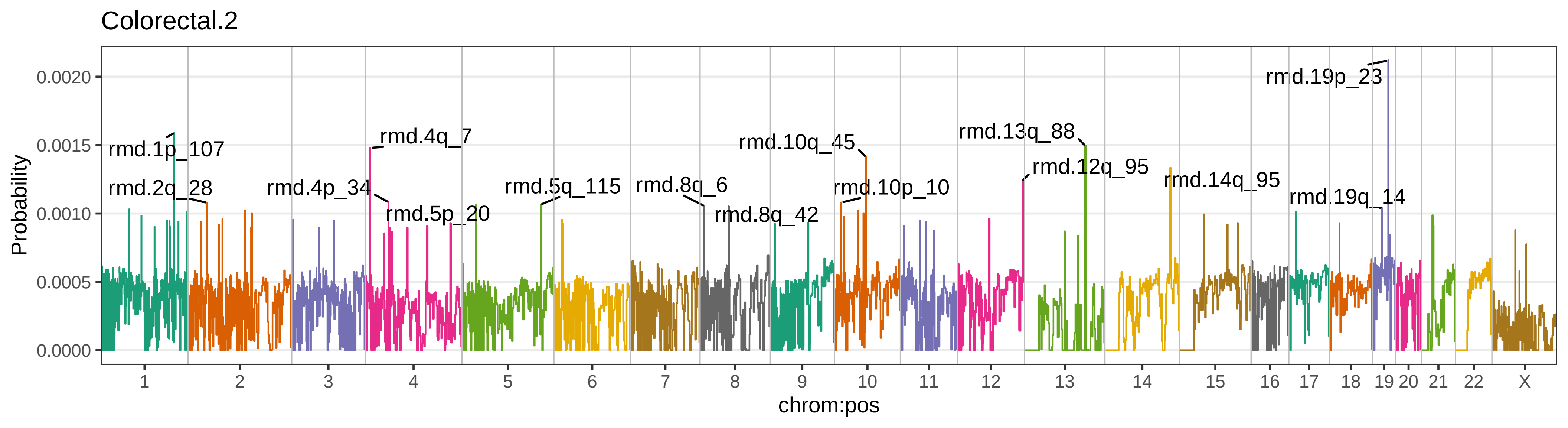

Colorectal.2
Probability
0.0020
0.0015
0.0010
0.0005
0.0000
rmd.1p_107
rmd.2q_28
rmd.4p_34
rmd.4q_7
rmd.5p_20
rmd.5q_115
rmd.8q_6
rmd.8q_42
rmd.10q_45
rmd.10p_10
rmd.13q_88
rmd.12q_95
rmd.14q_95
rmd.19p_23
rmd.19q_14
1
2
3
4
5
6
7
8
9
10
11
12
13
14
15
16
17
18
19
20
21
22
X
chrom:pos

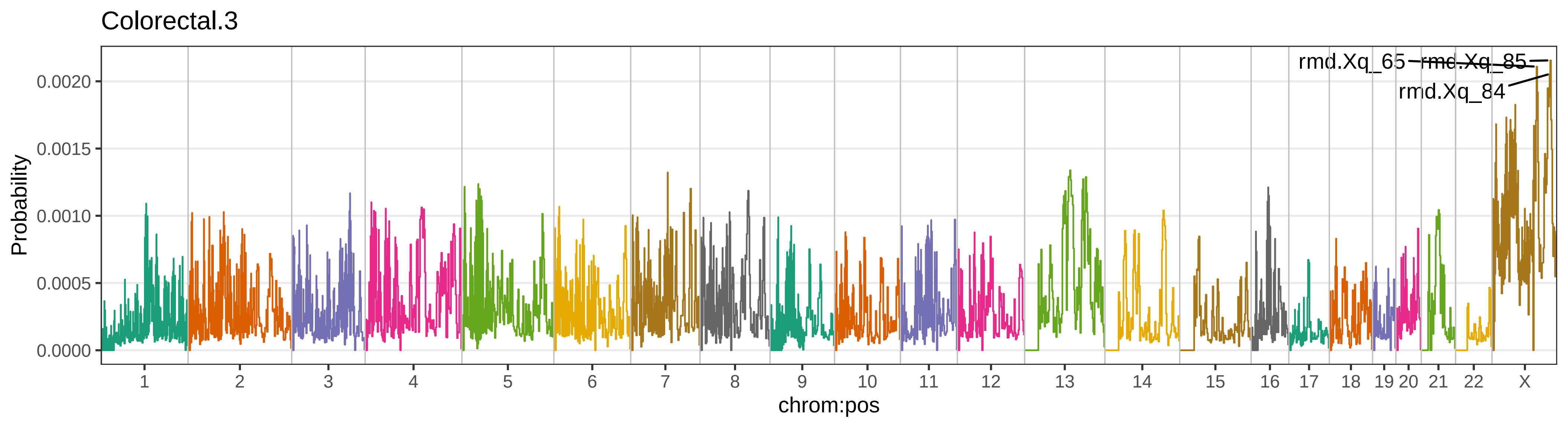

Colorectal.3
rmd.Xq_65
rmd.Xq_85
rmd.Xq_84
Probability
0.0020
0.0015
0.0010
0.0005
0.0000
1
2
3
4
5
6
7
8
9
10
11
12
13
14
15
16
17
18
19
20
21
22
X
chrom:pos

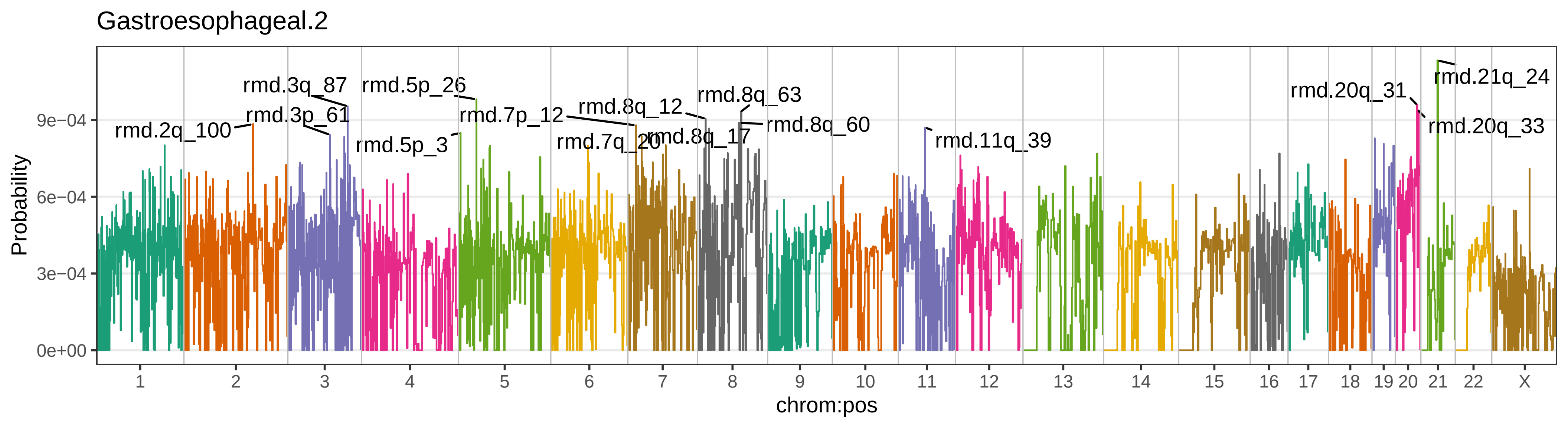

Gastroesophageal.2
Probability
9e−04
6e−04
3e−04
0e+00
rmd.2q_100
rmd.3q_87
rmd.3p_61
rmd.5p_26
rmd.5p_3
rmd.7p_12
rmd.7q_20
rmd.8q_12
rmd.8q_17
rmd.8q_63
rmd.8q_60
rmd.11q_39
rmd.20q_31
rmd.20q_33
rmd.21q_24
1
2
3
4
5
6
7
8
9
10
11
12
13
14
15
16
17
18
19
20
21
22
X
chrom:pos

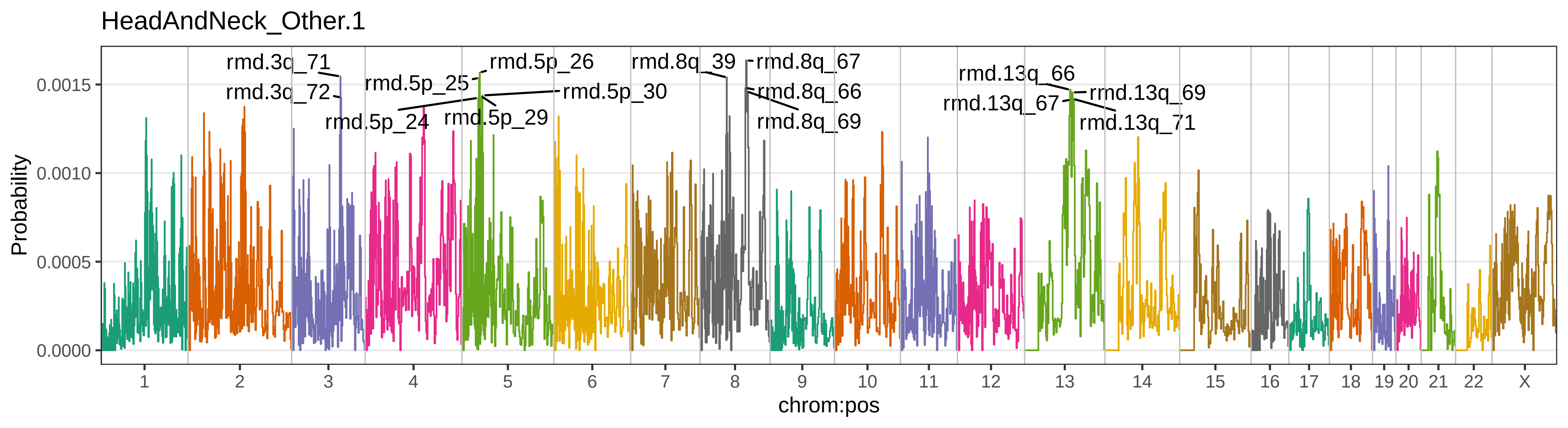
HeadAndNeck_Other.1
Probability
0.0015
0.0010
0.0005
0.0000
chrom:pos
1
2
3
4
5
6
7
8
9
10
11
12
13
14
15
16
17
18
19
20
21
22
X
rmd.3q_71
rmd.3q_72
rmd.5p_25
rmd.5p_26
rmd.5p_30
rmd.5p_24
rmd.5p_29
rmd.8q_39
rmd.8q_67
rmd.8q_66
rmd.8q_69
rmd.13q_66
rmd.13q_69
rmd.13q_67
rmd.13q_71

HeadAndNeck_Other.2

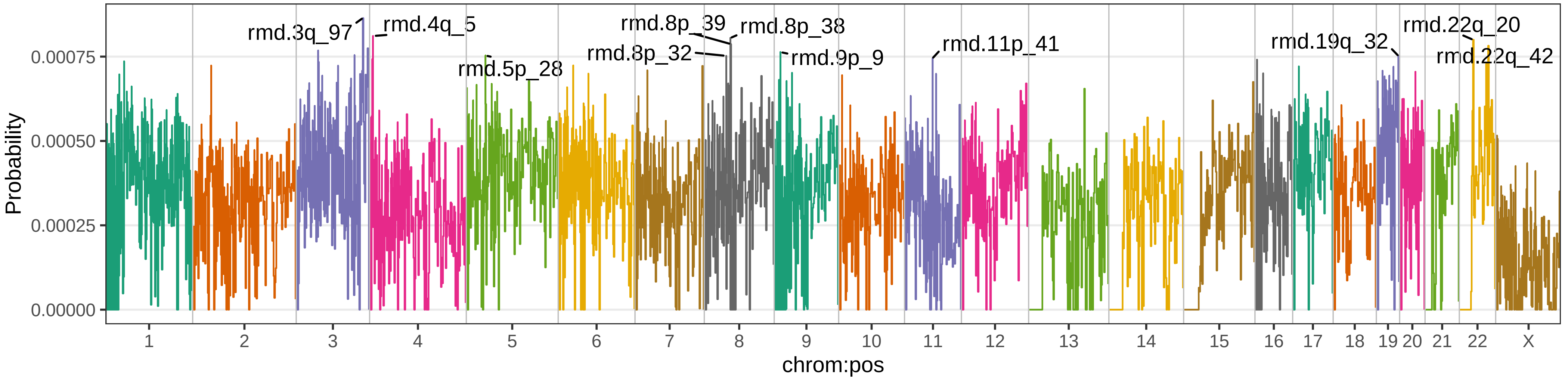

HeadAndNeck_SalivaryGland.1

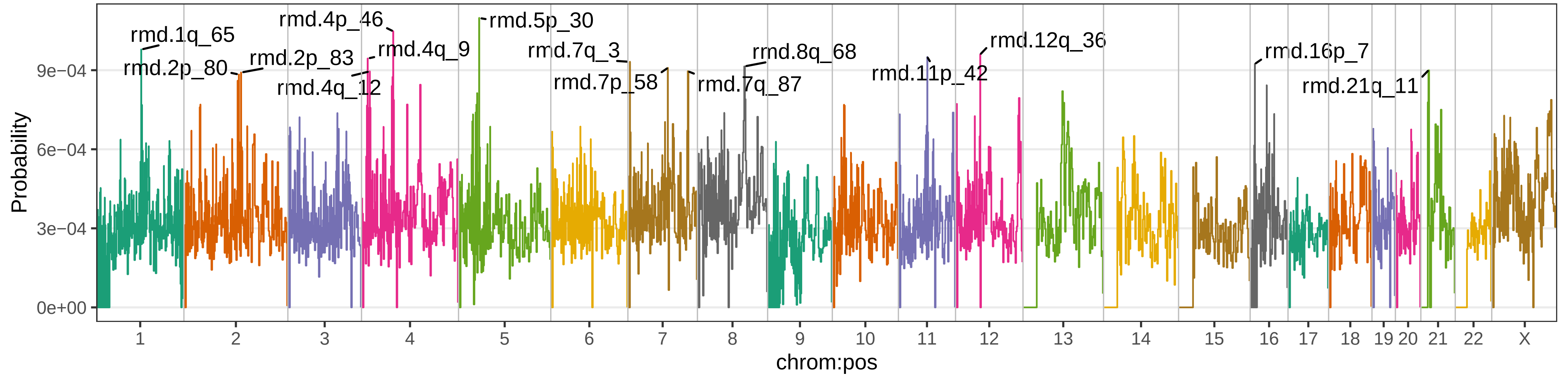

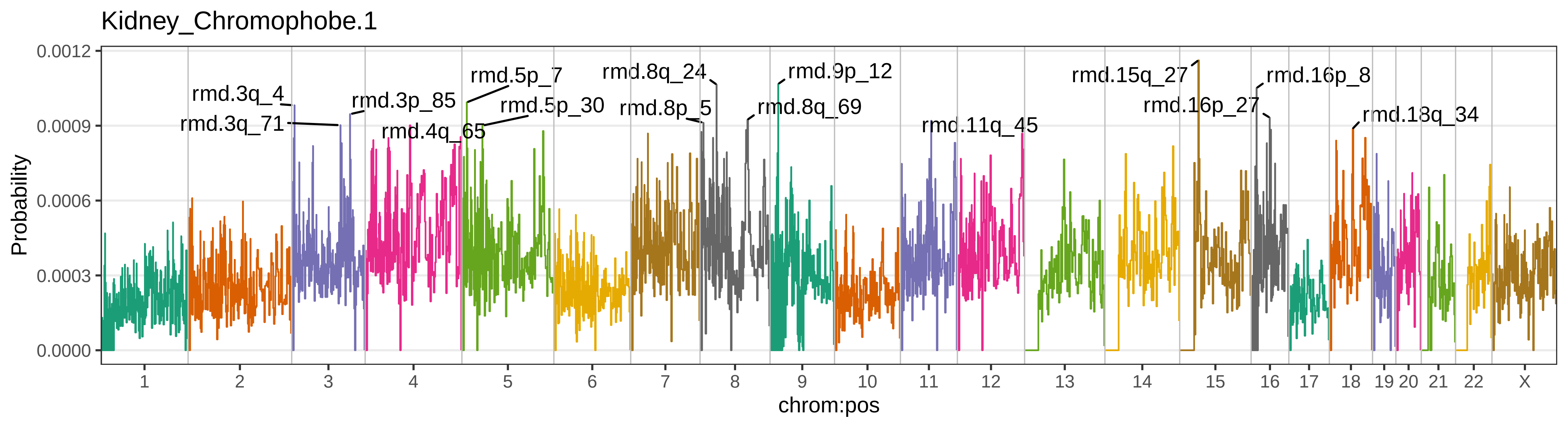
Kidney_Chromophobe.1
Probability
0.0012
0.0009
0.0006
0.0003
0.0000
rmd.3q_4
rmd.3q_71
rmd.3p_85
rmd.4q_65
rmd.5p_7
rmd.5p_30
rmd.8q_24
rmd.8p_5
rmd.9p_12
rmd.8q_69
rmd.11q_45
rmd.15q_27
rmd.16p_27
rmd.16p_8
rmd.18q_34
1 2 3 4 5 6 7 8 9 10 11 12 13 14 15 16 17 18 19 20 21 22 X
chrom:pos

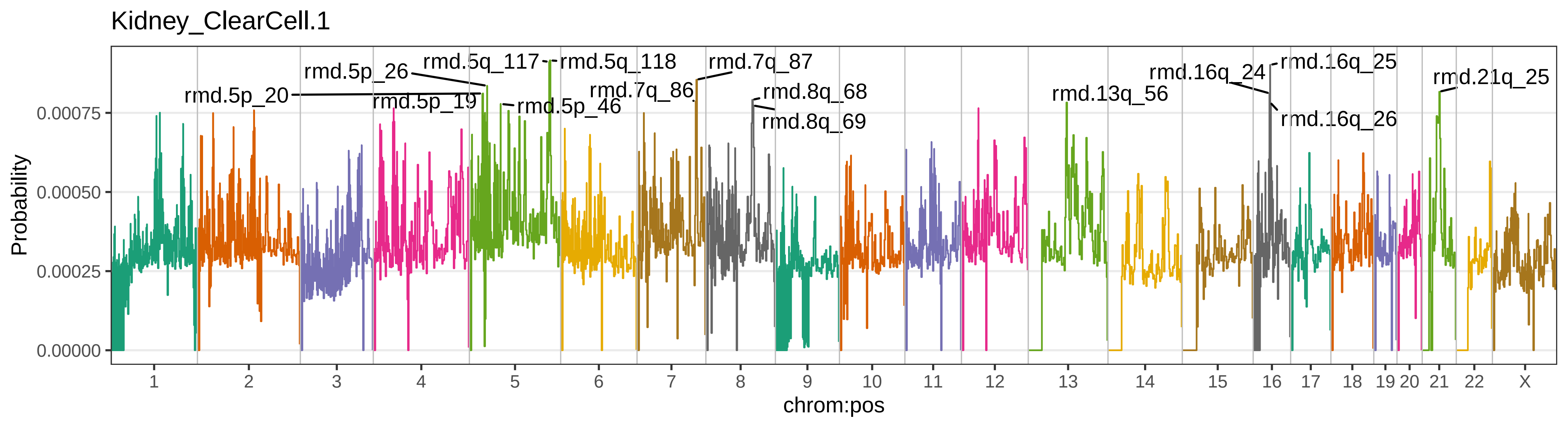

Kidney_ClearCell.1
Probability
0.00075
0.00050
0.00025
0.00000
rmd.5p_20
rmd.5p_26
rmd.5q_117
rmd.5q_118
rmd.5p_19
rmd.5p_46
rmd.7q_86
rmd.7q_87
rmd.8q_68
rmd.8q_69
rmd.13q_56
rmd.16q_24
rmd.16q_25
rmd.16q_26
rmd.21q_25
1
2
3
4
5
6
7
8
9
10
11
12
13
14
15
16
17
18
19
20
21
22
X
chrom:pos

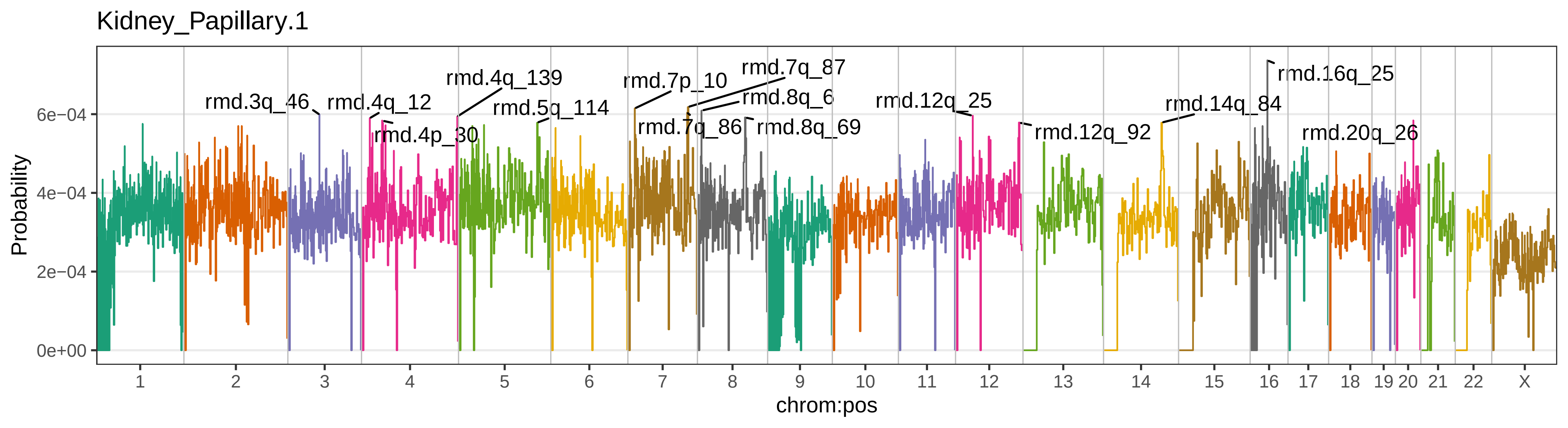
Kidney_Papillary.1
Probability
6e−04
4e−04
2e−04
0e+00
rmd.3q_46
rmd.4q_12
rmd.4p_30
rmd.4q_139
rmd.5q_114
rmd.7p_10
rmd.7q_87
rmd.8q_6
rmd.7q_86
rmd.8q_69
rmd.12q_25
rmd.12q_92
rmd.14q_84
rmd.16q_25
rmd.20q_26
1
2
3
4
5
6
7
8
9
10
11
12
13
14
15
16
17
18
19
20
21
22
X
chrom:pos

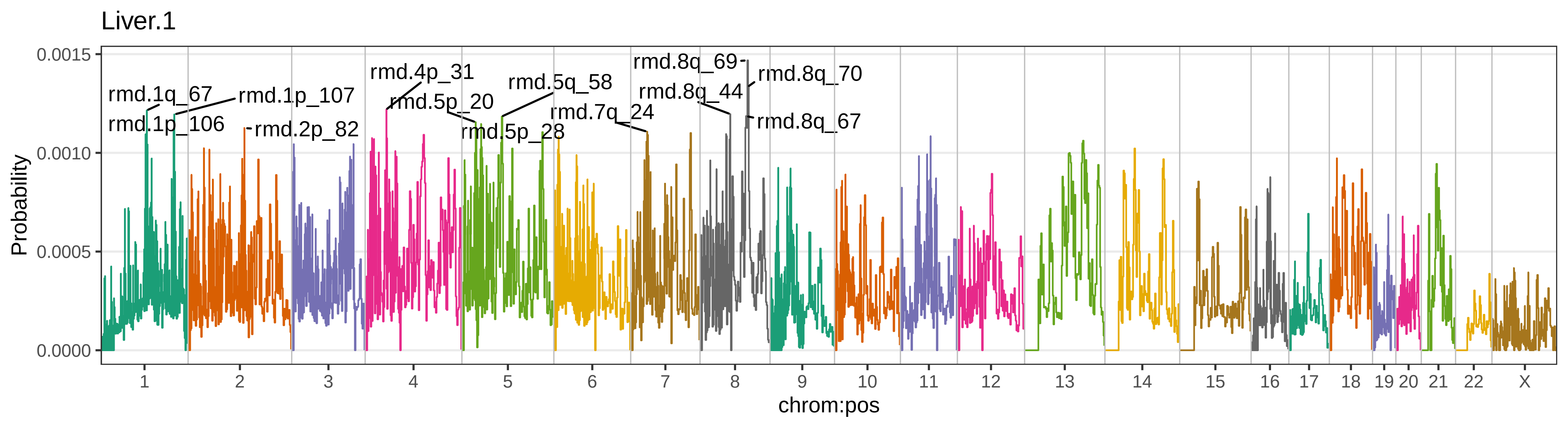

Liver.1
Probability
0.0015
0.0010
0.0005
0.0000
rmd.1q_67
rmd.1p_107
rmd.1p_106
rmd.2p_82
rmd.4p_31
rmd.5p_20
rmd.5p_28
rmd.5q_58
rmd.7q_24
rmd.8q_69
rmd.8q_44
rmd.8q_70
rmd.8q_67
1
2
3
4
5
6
7
8
9
10
11
12
13
14
15
16
17
18
19
20
21
22
X
chrom:pos

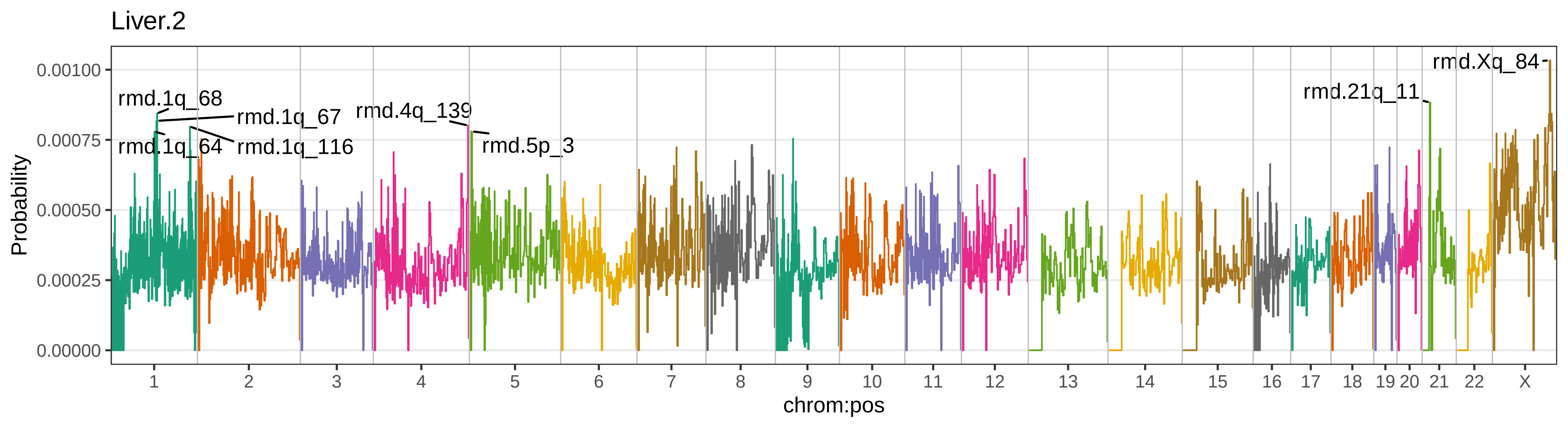
Liver.2
Probability
0.00100
0.00075
0.00050
0.00025
0.00000
rmd.1q_68
rmd.1q_67
rmd.1q_64
rmd.1q_116
rmd.4q_139
rmd.5p_3
rmd.21q_11
rmd.Xq_84
1 2 3 4 5 6 7 8 9 10 11 12 13 14 15 16 17 18 19 20 21 22 X
chrom:pos

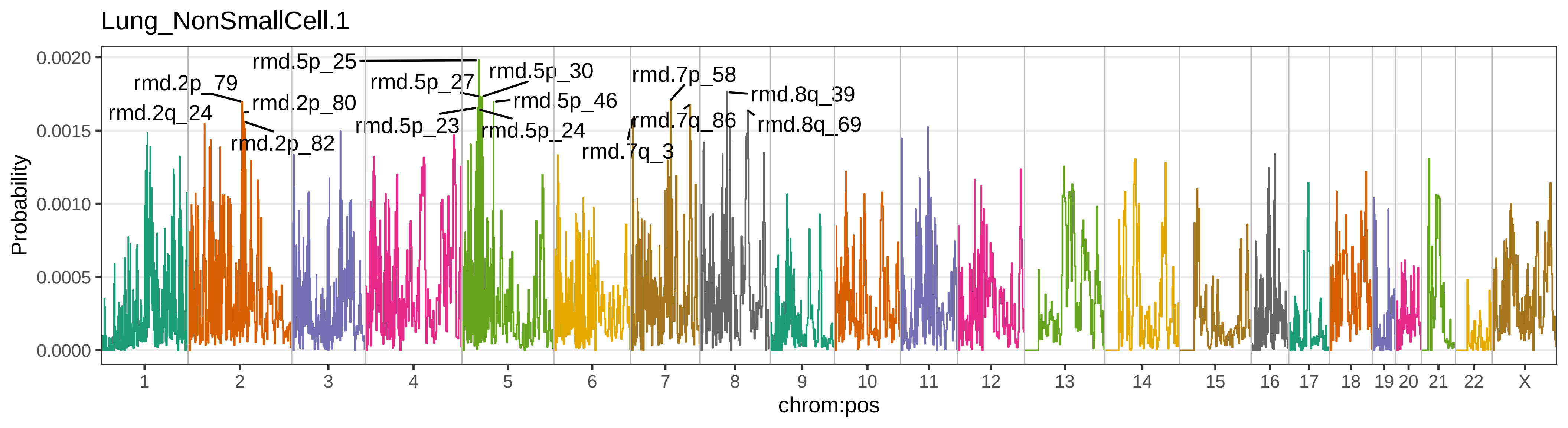
Lung_NonSmallCell.1
Probability
0.0020
0.0015
0.0010
0.0005
0.0000
rmd.5p_25
rmd.5p_30
rmd.5p_27
rmd.2p_79
rmd.2p_80
rmd.5p_46
rmd.2q_24
rmd.5p_23
rmd.5p_24
rmd.2p_82
rmd.7p_58
rmd.8q_39
rmd.7q_86
rmd.8q_69
rmd.7q_3
1
2
3
4
5
6
7
8
9
10
11
12
13
14
15
16
17
18
19
20
21
22
X
chrom:pos

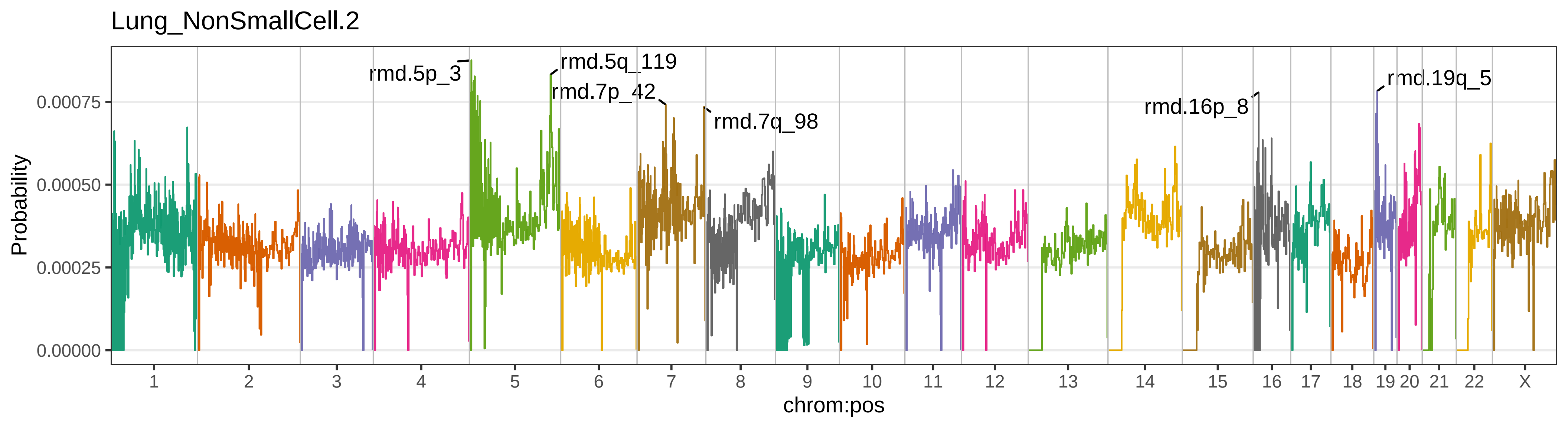

Lung_NonSmallCell.2
rmd.5p_3
rmd.5q_119
rmd.7p_42
rmd.7q_98
rmd.16p_8
rmd.19q_5
0.00075
0.00050
0.00025
0.00000
Probability
1
2
3
4
5
6
7
8
9
10
11
12
13
14
15
16
17
18
19
20
21
22
X
chrom:pos

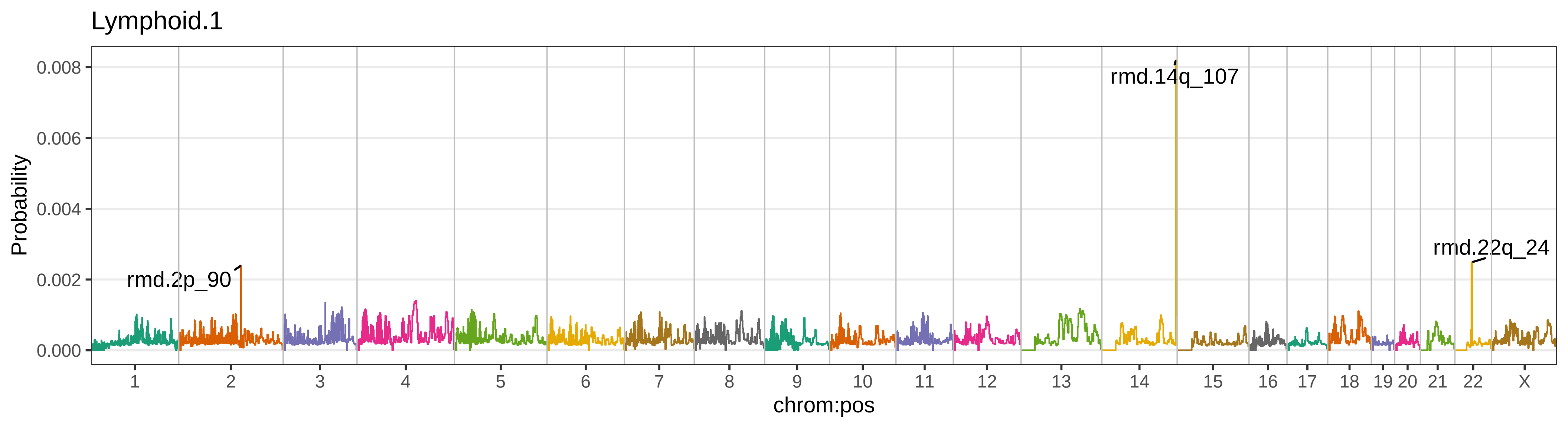
Lymphoid.1
Probability
0.008
0.006
0.004
0.002
0.000
rmd.2p_90
rmd.14q_107
rmd.22q_24
1
2
3
4
5
6
7
8
9
10
11
12
13
14
15
16
17
18
19
20
21
22
X
chrom:pos

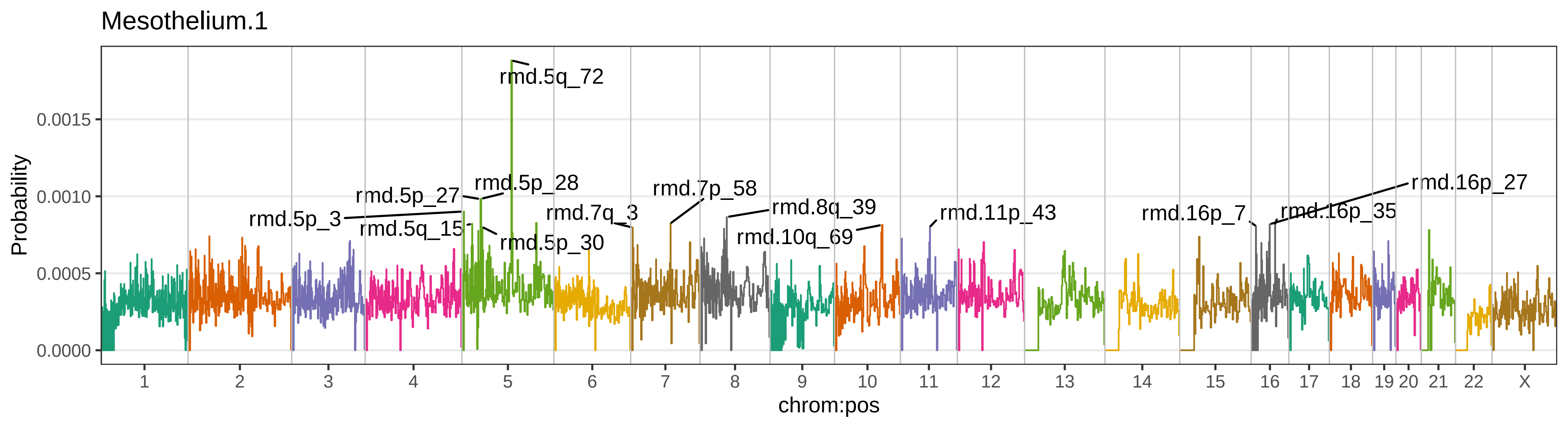

Mesothelium.1
Probability
0.0015
0.0010
0.0005
0.0000
rmd.5q_72
rmd.5p_28
rmd.5p_27
rmd.5p_3
rmd.5q_15
rmd.5p_30
rmd.7q_3
rmd.7p_58
rmd.8q_39
rmd.10q_69
rmd.11p_43
rmd.16p_7
rmd.16p_35
rmd.16p_27
1
2
3
4
5
6
7
8
9
10
11
12
13
14
15
16
17
18
19
20
21
22
X
chrom:pos

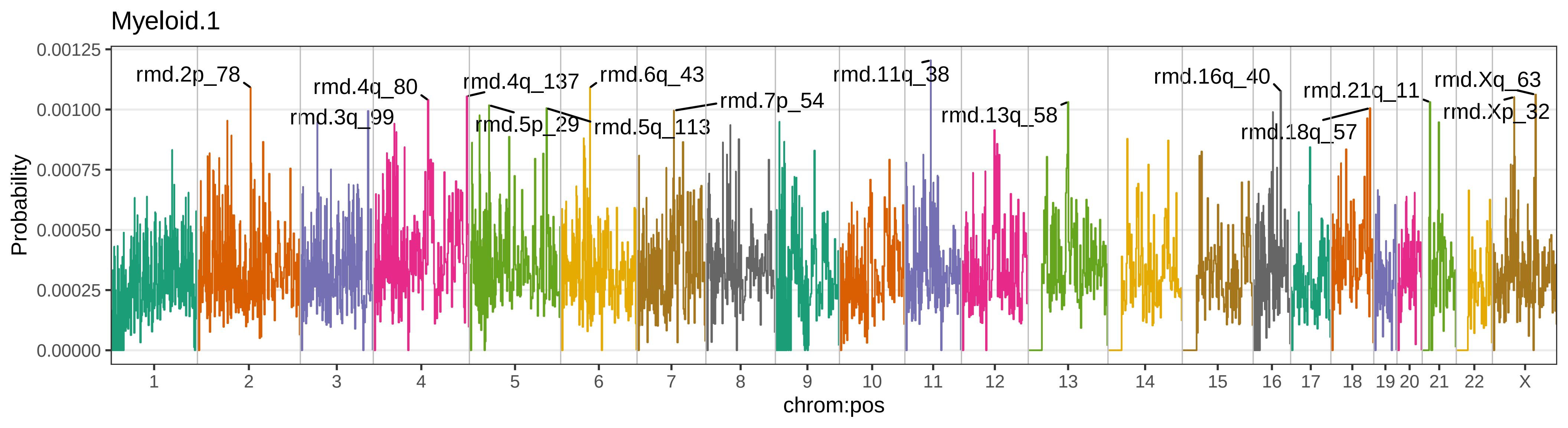
Myeloid.1
Probability
0.00125
0.00100
0.00075
0.00050
0.00025
0.00000
rmd.2p_78
rmd.3q_99
rmd.4q_80
rmd.4q_137
rmd.5p_29
rmd.6q_43
rmd.5q_113
rmd.7p_54
rmd.11q_38
rmd.13q_58
rmd.16q_40
rmd.18q_57
rmd.21q_11
rmd.Xq_63
rmd.Xp_32
1 2 3 4 5 6 7 8 9 10 11 12 13 14 15 16 17 18 19 20 21 22 X
chrom:pos

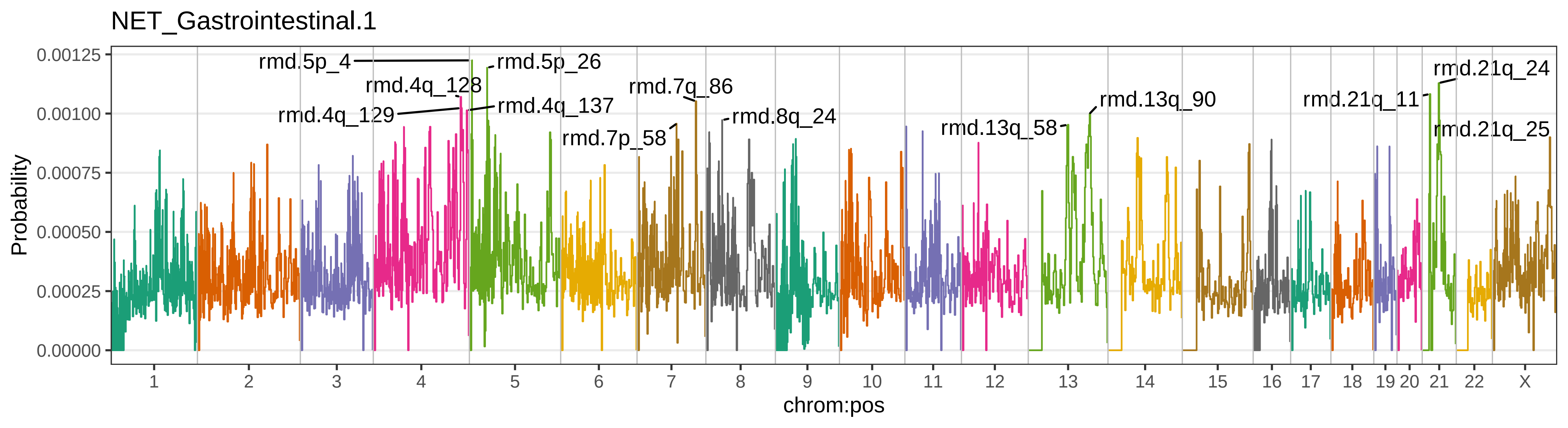

NET_Gastrointestinal.1
Probability
0.00125
0.00100
0.00075
0.00050
0.00025
0.00000
rmd.5p_4
rmd.5p_26
rmd.4q_128
rmd.4q_137
rmd.4q_129
rmd.7q_86
rmd.7p_58
rmd.8q_24
rmd.13q_58
rmd.13q_90
rmd.21q_11
rmd.21q_24
rmd.21q_25
1 2 3 4 5 6 7 8 9 10 11 12 13 14 15 16 17 18 19 20 21 22 X
chrom:pos

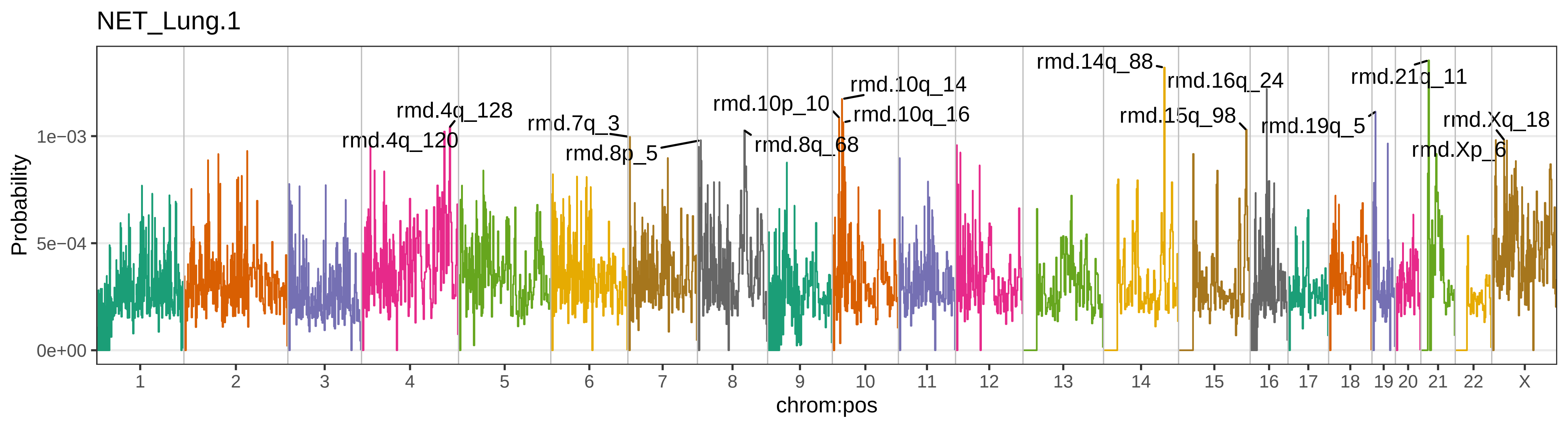

NET_Lung.1
Probability
1e−03
5e−04
0e+00
chrom:pos
1
2
3
4
5
6
7
8
9
10
11
12
13
14
15
16
17
18
19
20
21
22
X
rmd.4q_128
rmd.4q_120
rmd.7q_3
rmd.8p_5
rmd.10p_10
rmd.10q_14
rmd.10q_16
rmd.8q_68
rmd.14q_88
rmd.16q_24
rmd.15q_98
rmd.19q_5
rmd.21q_11
rmd.Xq_18
rmd.Xp_6

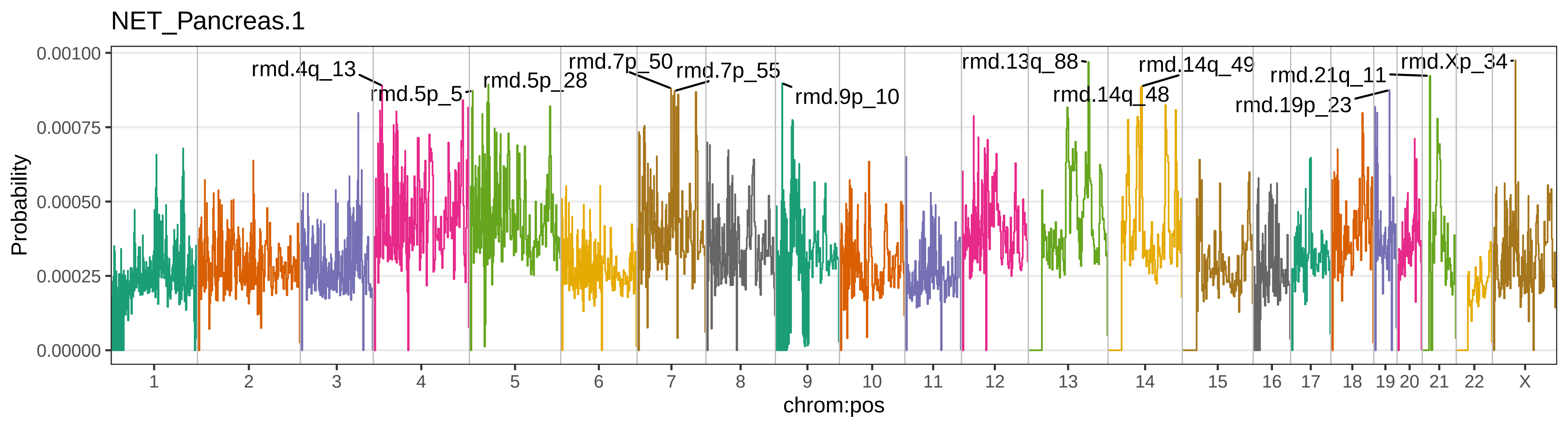

NET_Pancreas.1
Probability
0.00100
0.00075
0.00050
0.00025
0.00000
rmd.4q_13
rmd.5p_5
rmd.5p_28
rmd.7p_50
rmd.7p_55
rmd.9p_10
rmd.13q_88
rmd.14q_48
rmd.14q_49
rmd.19p_23
rmd.21q_11
rmd.Xp_34
1
2
3
4
5
6
7
8
9
10
11
12
13
14
15
16
17
18
19
20
21
22
X
chrom:pos

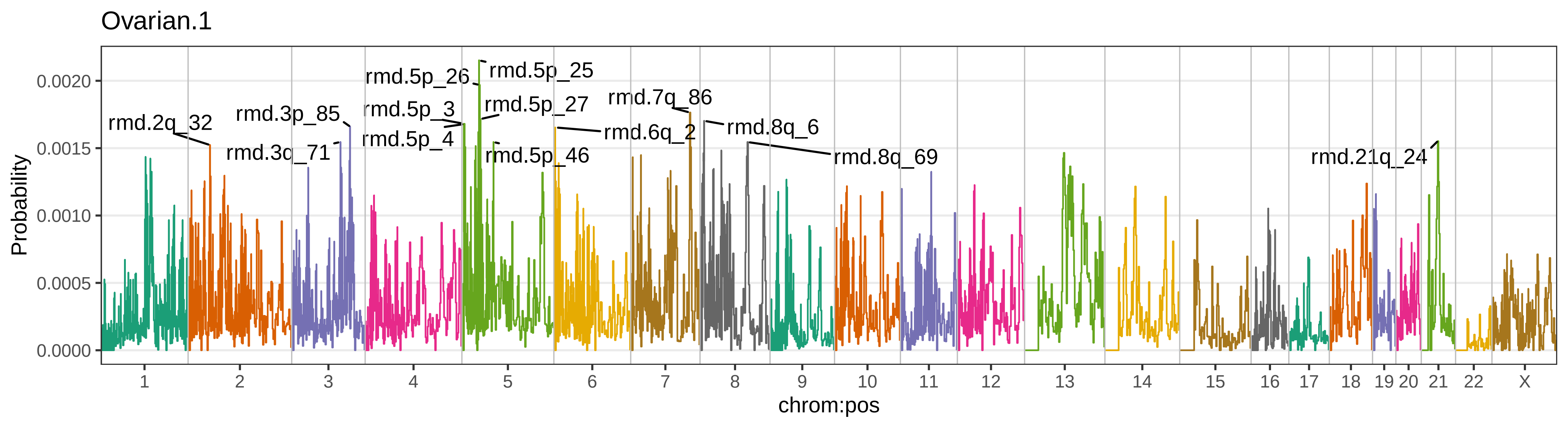

Ovarian.1
Probability
0.0020
0.0015
0.0010
0.0005
0.0000
rmd.2q_32
rmd.3p_85
rmd.3q_71
rmd.5p_26
rmd.5p_25
rmd.5p_3
rmd.5p_4
rmd.5p_27
rmd.5p_46
rmd.7q_86
rmd.6q_2
rmd.8q_6
rmd.8q_69
rmd.21q_24
1
2
3
4
5
6
7
8
9
10
11
12
13
14
15
16
17
18
19
20
21
22
X
chrom:pos

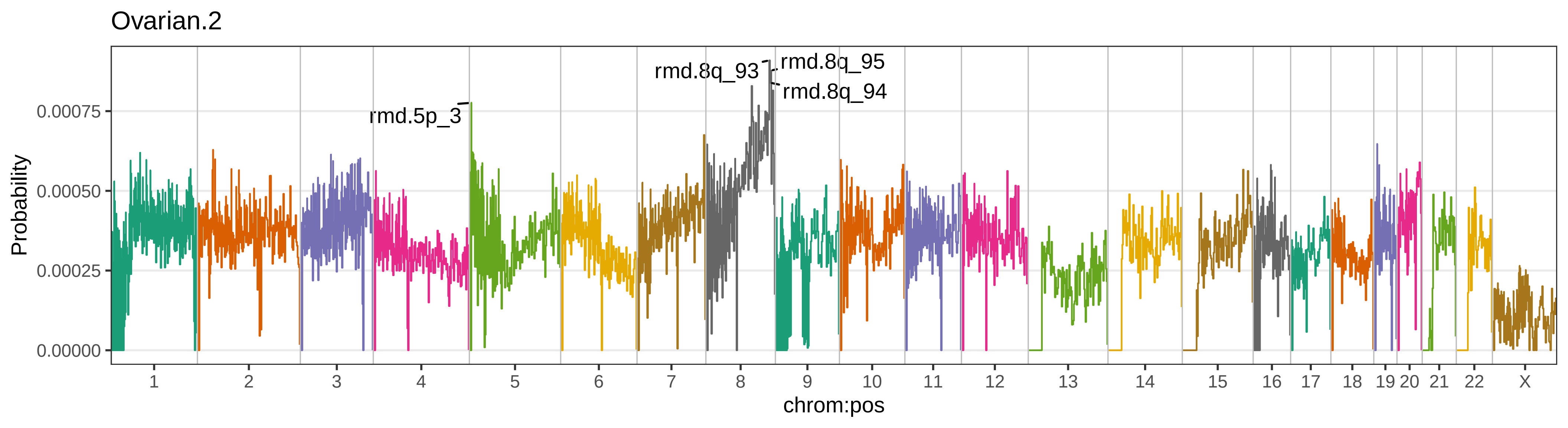

Ovarian.2
rmd.8q_95
rmd.8q_93
rmd.8q_94
rmd.5p_3
0.00075
0.00050
0.00025
0.00000
Probability
1
2
3
4
5
6
7
8
9
10
11
12
13
14
15
16
17
18
19
20
21
22
X
chrom:pos

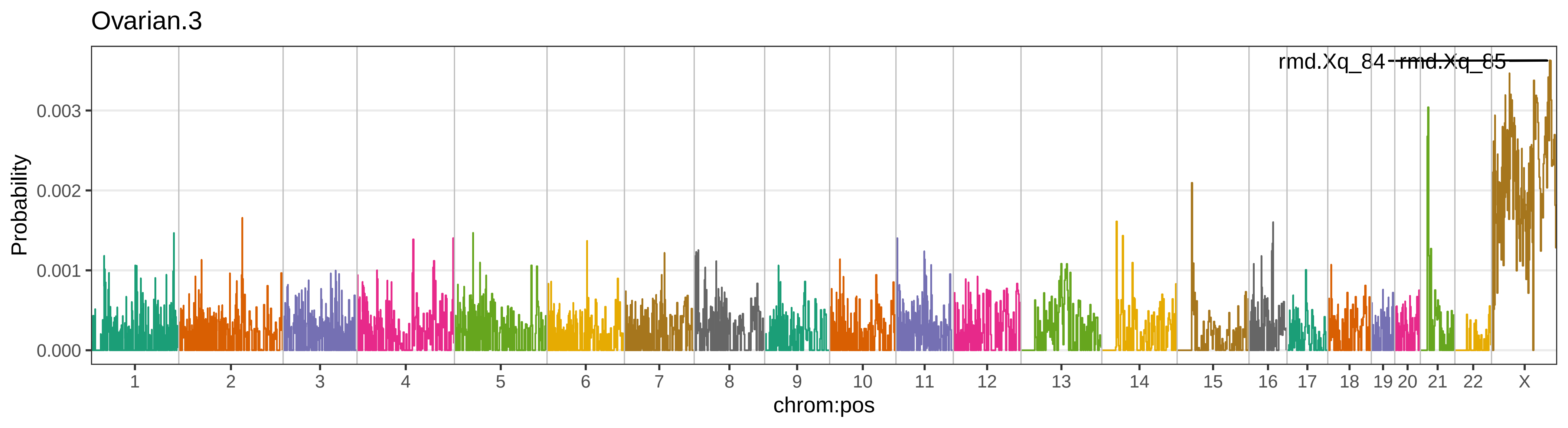
Ovarian.3
rmd.Xq_84
rmd.Xq_85
Probability
0.000
0.001
0.002
0.003
1
2
3
4
5
6
7
8
9
10
11
12
13
14
15
16
17
18
19
20
21
22
X
chrom:pos

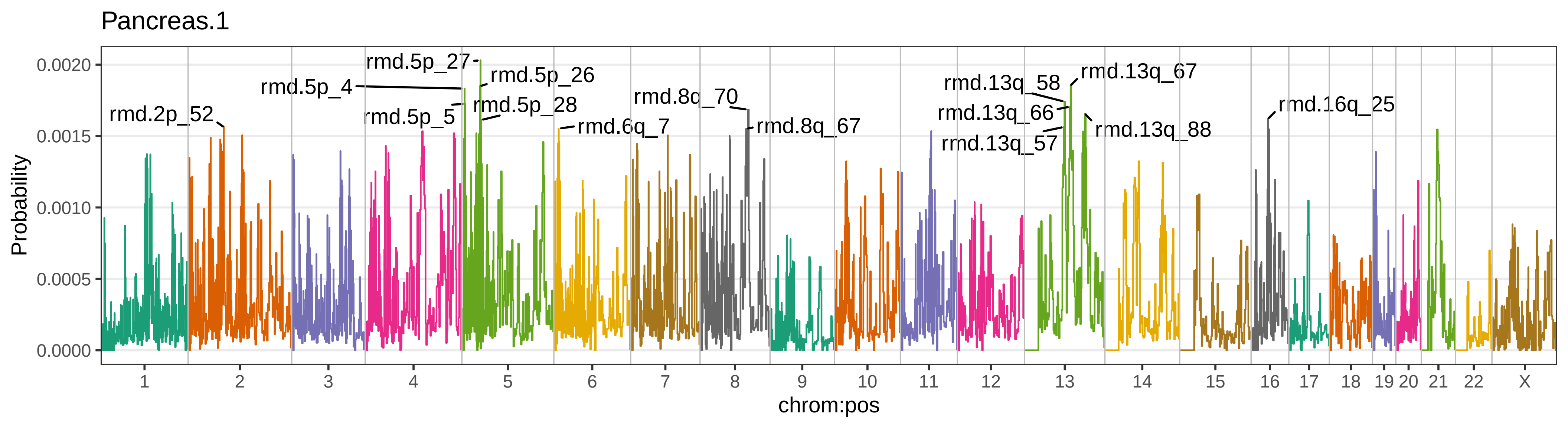

Pancreas.1
Probability
0.0020
0.0015
0.0010
0.0005
0.0000
rmd.2p_52
rmd.5p_4
rmd.5p_27
rmd.5p_26
rmd.5p_5
rmd.5p_28
rmd.6q_7
rmd.8q_70
rmd.8q_67
rmd.13q_58
rmd.13q_67
rmd.13q_66
rmd.13q_57
rmd.13q_88
rmd.16q_25
1
2
3
4
5
6
7
8
9
10
11
12
13
14
15
16
17
18
19
20
21
22
X
chrom:pos

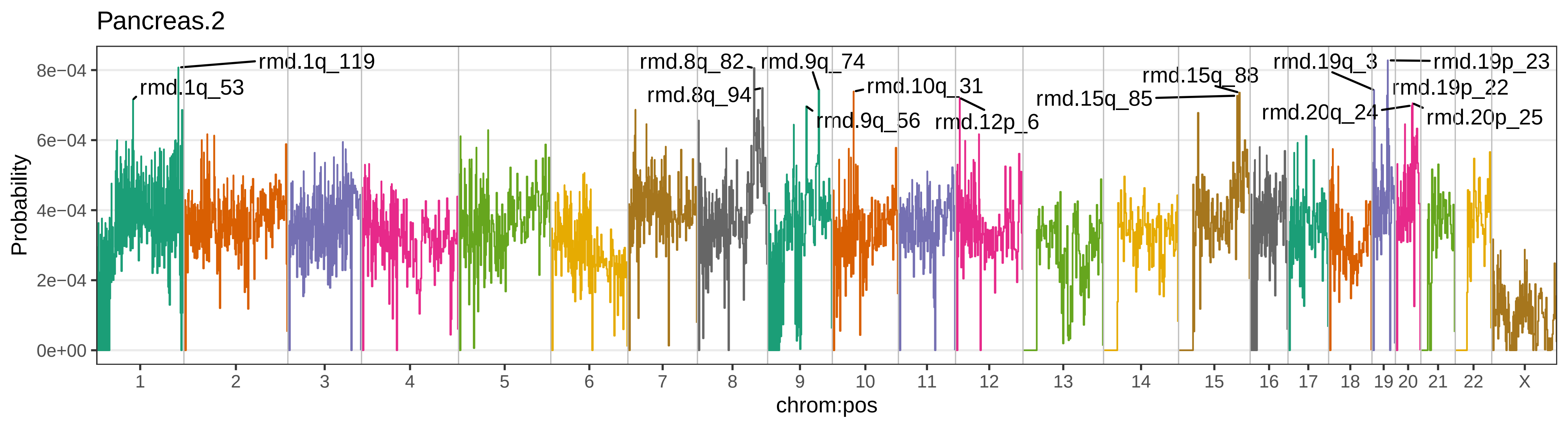

Pancreas.2
Probability
chrom:pos
rmd.1q_119
rmd.1q_53
rmd.8q_82
rmd.9q_74
rmd.8q_94
rmd.9q_56
rmd.10q_31
rmd.12p_6
rmd.15q_88
rmd.15q_85
rmd.19q_3
rmd.19p_23
rmd.19p_22
rmd.20q_24
rmd.20p_25
8e−04
6e−04
4e−04
2e−04
0e+00
1
2
3
4
5
6
7
8
9
10
11
12
13
14
15
16
17
18
19
20
21
22
X

Pancreas.3

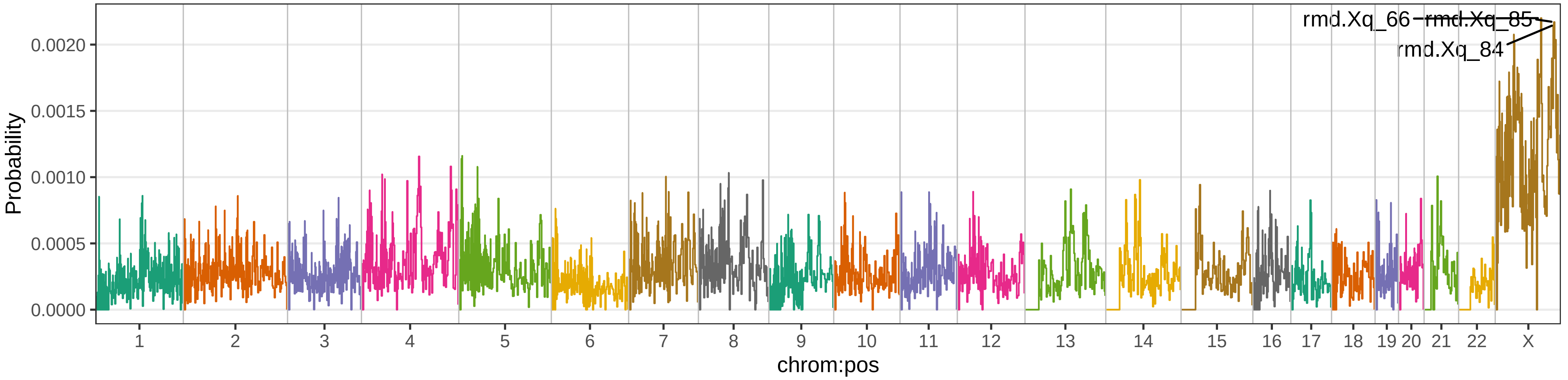

rmd.Xq_66
rmd.Xq_85
rmd.Xq_84
Probability
0.0020
0.0015
0.0010
0.0005
0.0000
1 2 3 4 5 6 7 8 9 10 11 12 13 14 15 16 17 18 19 20 21 22 X
chrom:pos

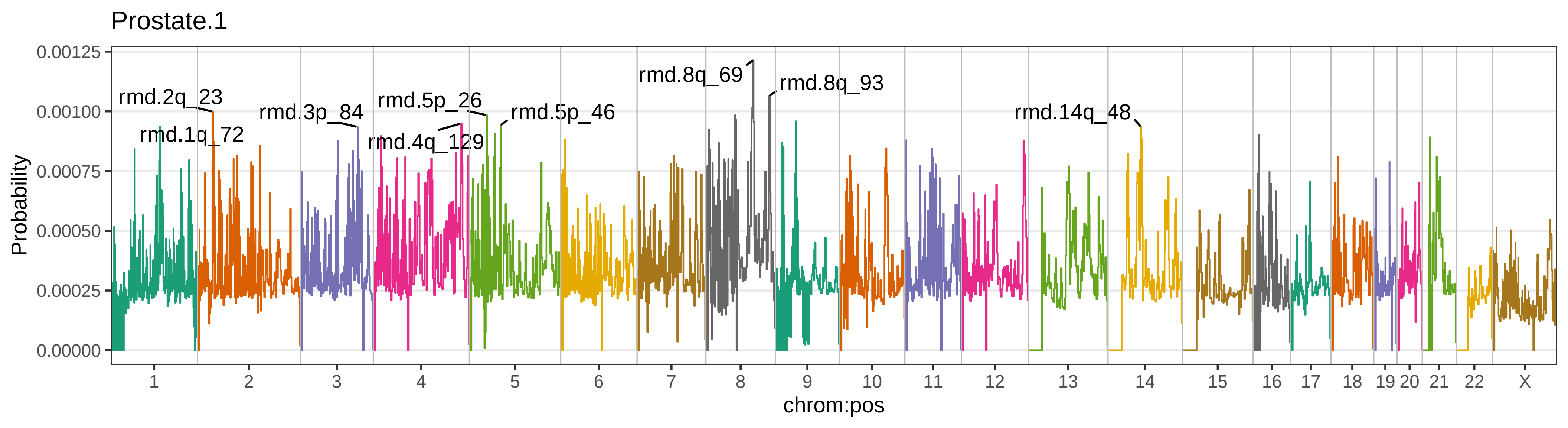
Prostate.1
Probability
0.00125
0.00100
0.00075
0.00050
0.00025
0.00000
rmd.2q_23
rmd.1q_72
rmd.3p_84
rmd.5p_26
rmd.4q_129
rmd.5p_46
rmd.8q_69
rmd.8q_93
rmd.14q_48
1
2
3
4
5
6
7
8
9
10
11
12
13
14
15
16
17
18
19
20
21
22
X
chrom:pos

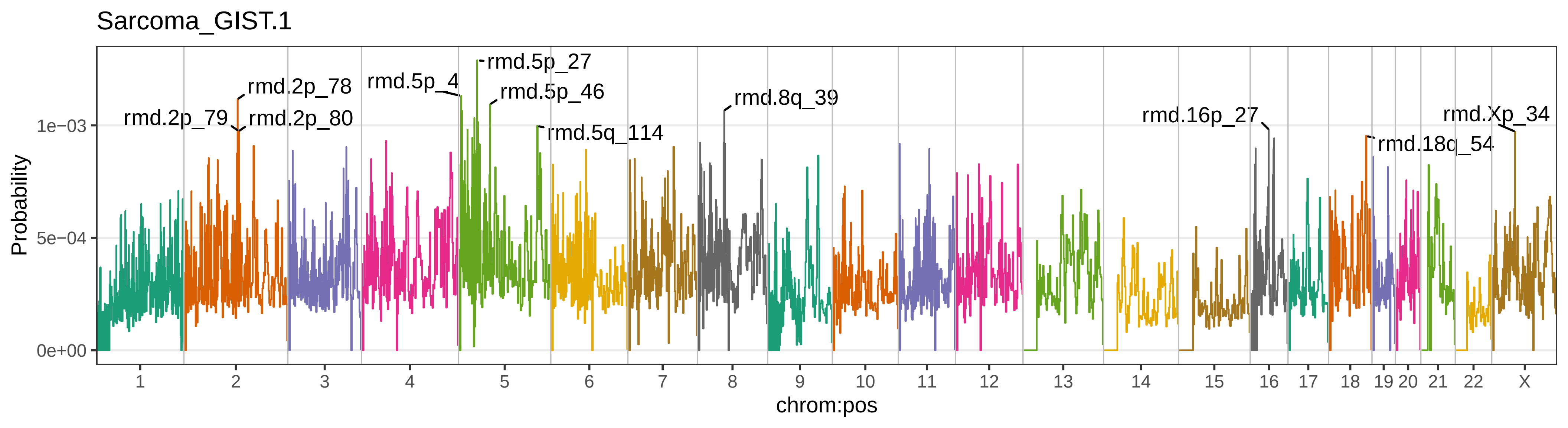

Sarcoma_GIST.1
Probability
1e−03
5e−04
0e+00
rmd.2p_79
rmd.2p_78
rmd.2p_80
rmd.5p_4
rmd.5p_27
rmd.5p_46
rmd.5q_114
rmd.8q_39
rmd.16p_27
rmd.18q_54
rmd.Xp_34
1
2
3
4
5
6
7
8
9
10
11
12
13
14
15
16
17
18
19
20
21
22
X
chrom:pos

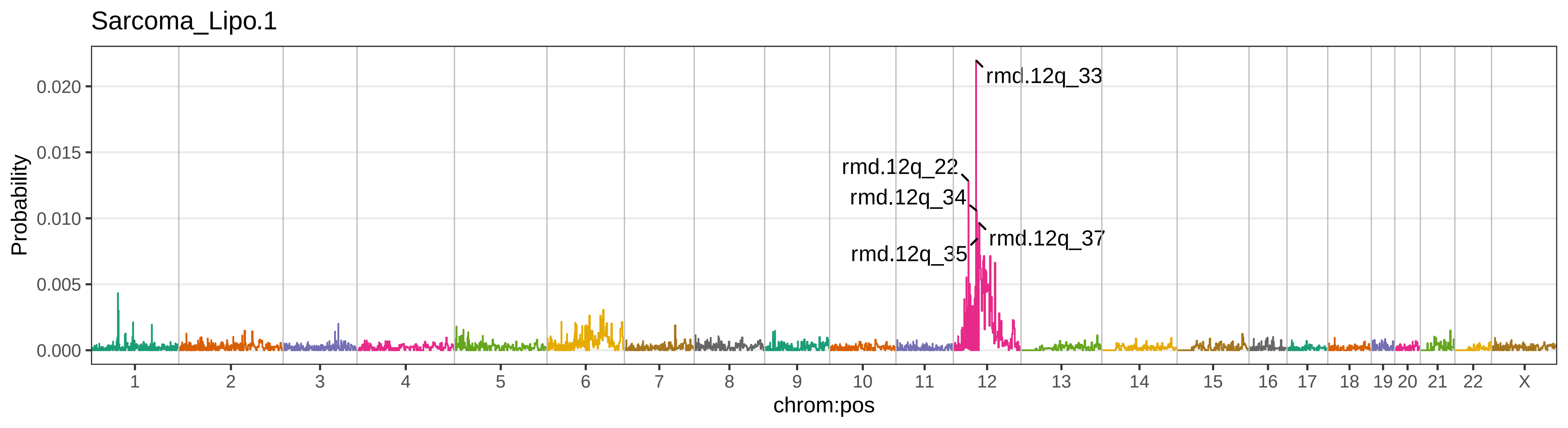

Sarcoma_Lipo.1
Probability
0.020
0.015
0.010
0.005
0.000
rmd.12q_33
rmd.12q_22
rmd.12q_34
rmd.12q_37
rmd.12q_35
1
2
3
4
5
6
7
8
9
10
11
12
13
14
15
16
17
18
19
20
21
22
X
chrom:pos

Sarcoma_Lipo.2

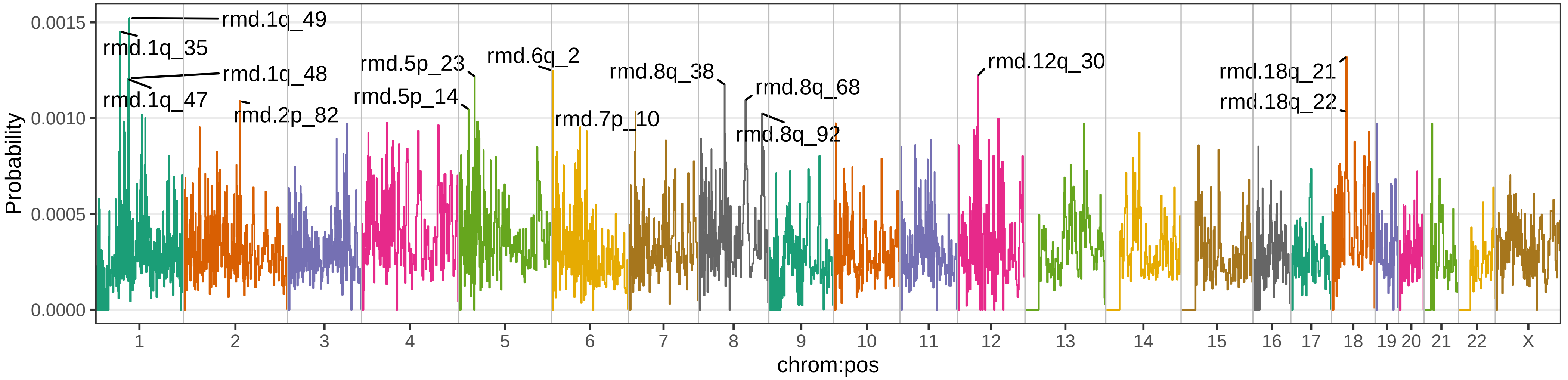

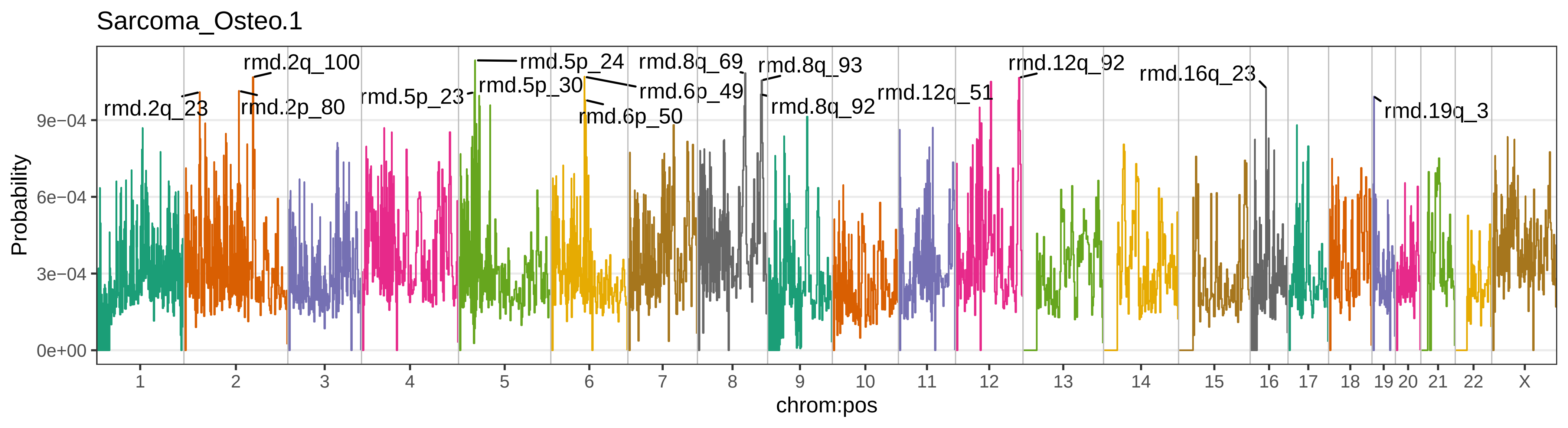

Sarcoma_Osteo.1
rmd.2q_100
rmd.5p_24
rmd.8q_69
rmd.8q_93
rmd.12q_92
rmd.16q_23
rmd.5p_30
rmd.6p_49
rmd.12q_51
rmd.5p_23
rmd.2q_23
rmd.2p_80
rmd.8q_92
rmd.19q_3
rmd.6p_50
Probability
9e−04
6e−04
3e−04
0e+00
1 2 3 4 5 6 7 8 9 10 11 12 13 14 15 16 17 18 19 20 21 22 X
chrom:pos

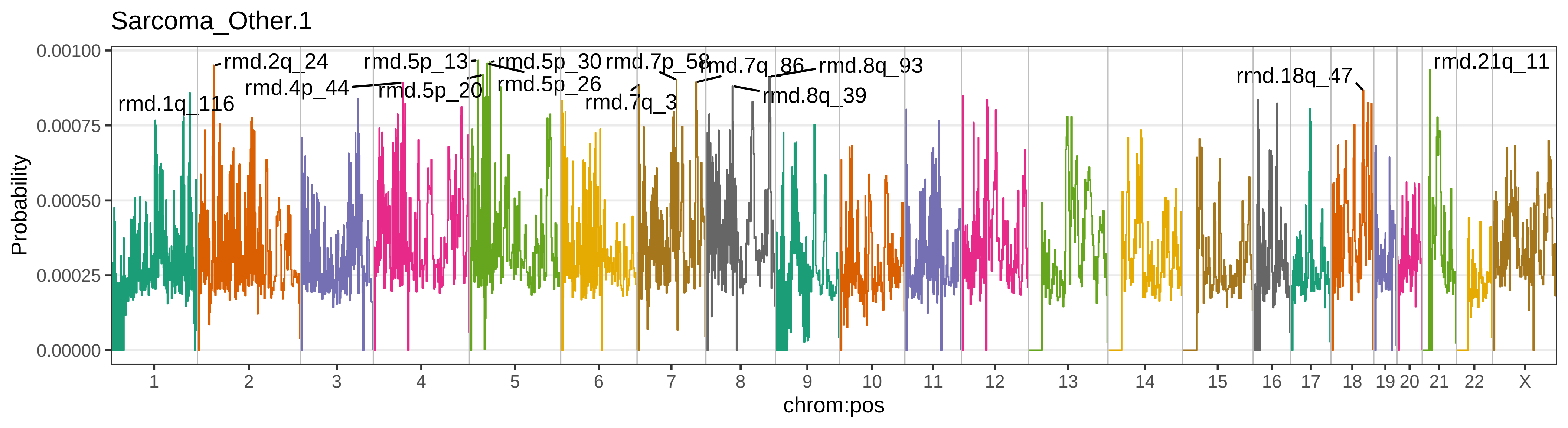
Sarcoma_Other.1
Probability
chrom:pos
0.00100
0.00075
0.00050
0.00025
0.00000
1
2
3
4
5
6
7
8
9
10
11
12
13
14
15
16
17
18
19
20
21
22
X
rmd.1q_116
rmd.2q_24
rmd.4p_44
rmd.5p_13
rmd.5p_20
rmd.5p_30
rmd.5p_26
rmd.7p_58
rmd.7q_3
rmd.7q_86
rmd.8q_93
rmd.8q_39
rmd.18q_47
rmd.21q_11

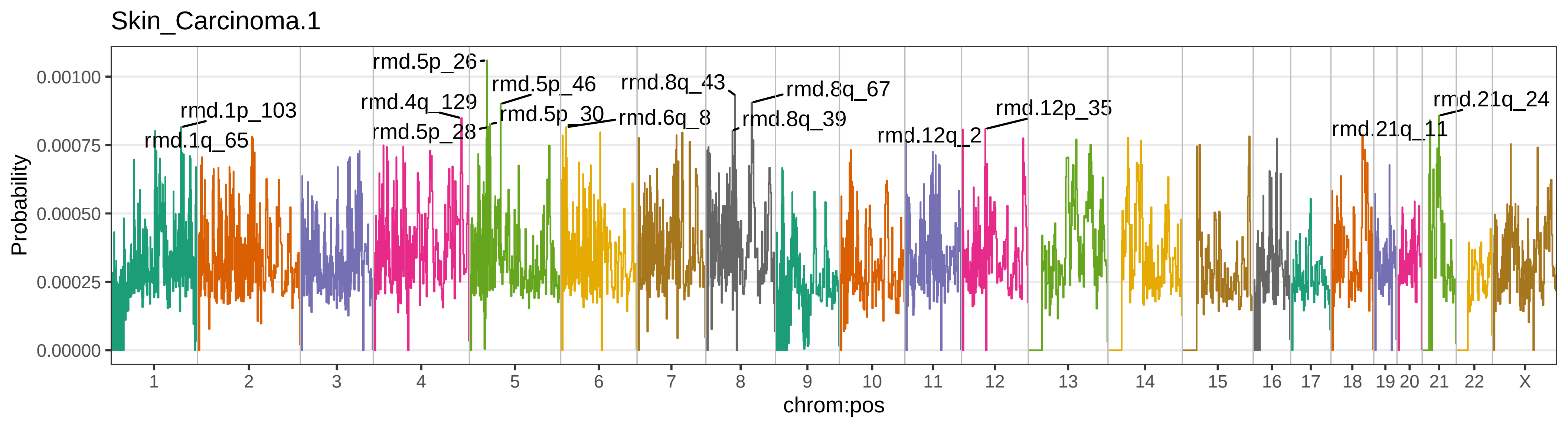
Skin_Carcinoma.1
Probability
chrom:pos
0.00100
0.00075
0.00050
0.00025
0.00000
1
2
3
4
5
6
7
8
9
10
11
12
13
14
15
16
17
18
19
20
21
22
X
rmd.5p_26
rmd.5p_46
rmd.8q_43
rmd.8q_67
rmd.1p_103
rmd.4q_129
rmd.5p_30
rmd.6q_8
rmd.8q_39
rmd.12p_35
rmd.21q_24
rmd.1q_65
rmd.5p_28
rmd.12q_2
rmd.21q_11

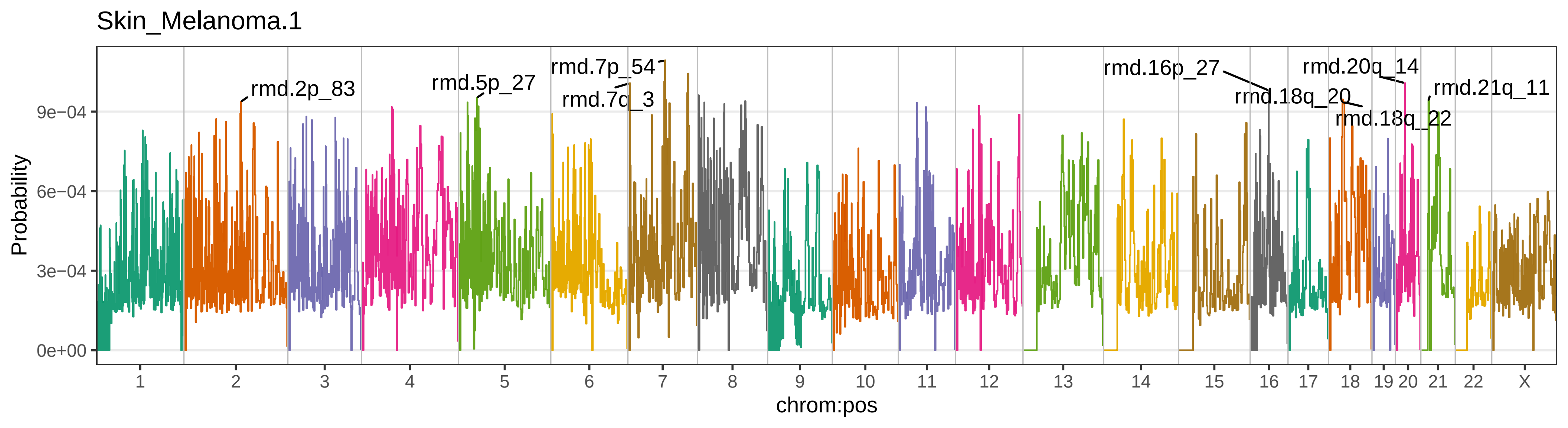

Skin_Melanoma.1
rmd.2p_83
rmd.5p_27
rmd.7p_54
rmd.7q_3
rmd.16p_27
rmd.18q_20
rmd.18q_22
rmd.20q_14
rmd.21q_11
Probability
9e−04
6e−04
3e−04
0e+00
1
2
3
4
5
6
7
8
9
10
11
12
13
14
15
16
17
18
19
20
21
22
X
chrom:pos

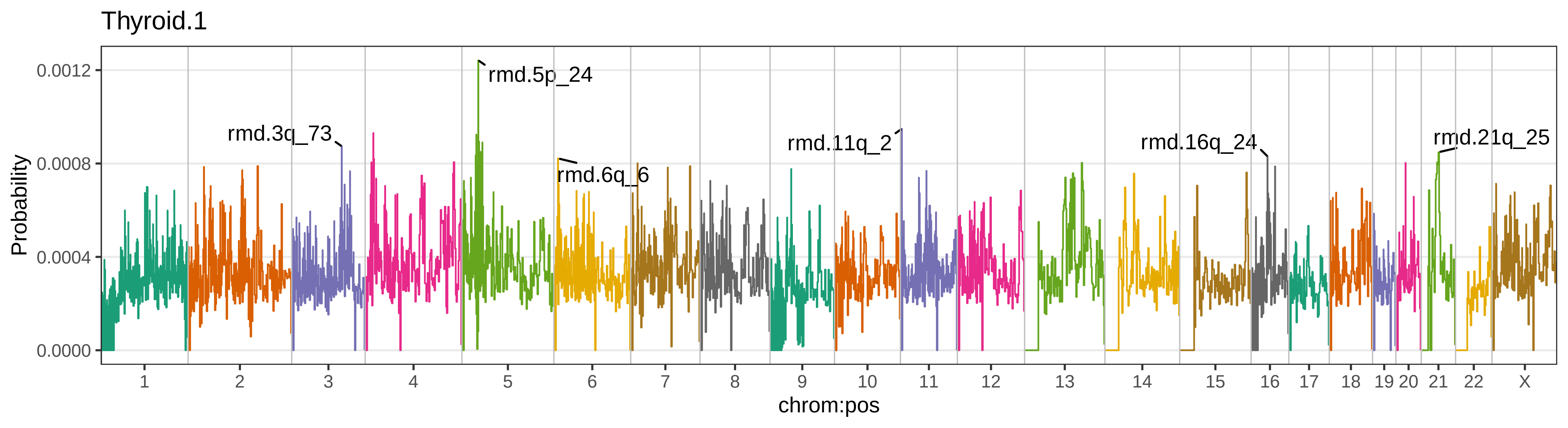

Thyroid.1
Probability
0.0012
0.0008
0.0004
0.0000
rmd.5p_24
rmd.3q_73
rmd.6q_6
rmd.11q_2
rmd.16q_24
rmd.21q_25
1
2
3
4
5
6
7
8
9
10
11
12
13
14
15
16
17
18
19
20
21
22
X
chrom:pos

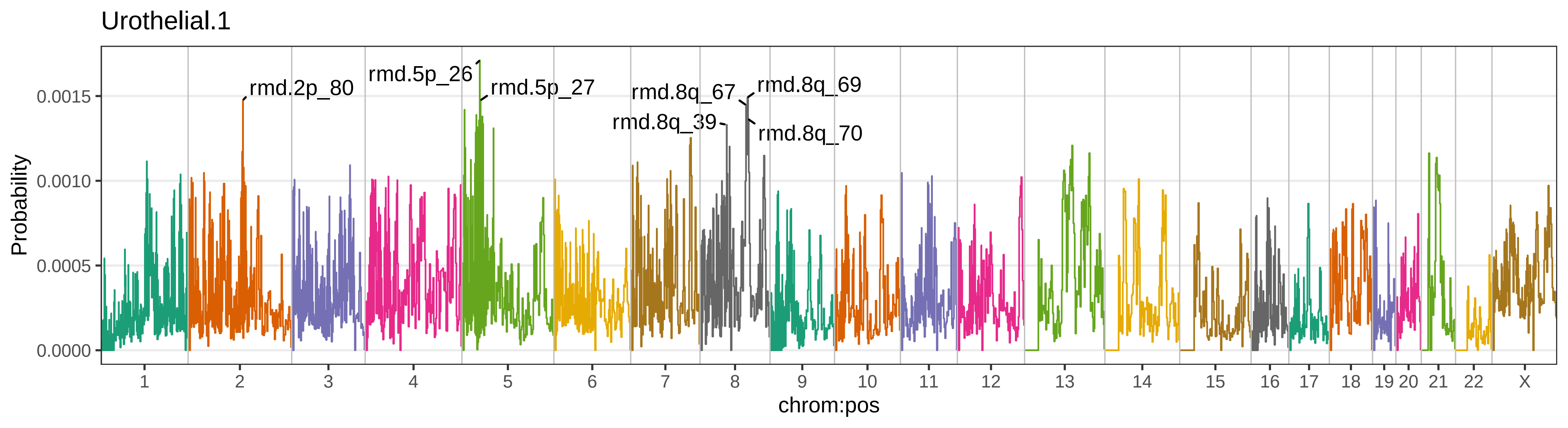
Urothelial.1
Probability
0.0015
0.0010
0.0005
0.0000
rmd.2p_80
rmd.5p_26
rmd.5p_27
rmd.8q_67
rmd.8q_69
rmd.8q_39
rmd.8q_70
1
2
3
4
5
6
7
8
9
10
11
12
13
14
15
16
17
18
19
20
21
22
X
chrom:pos

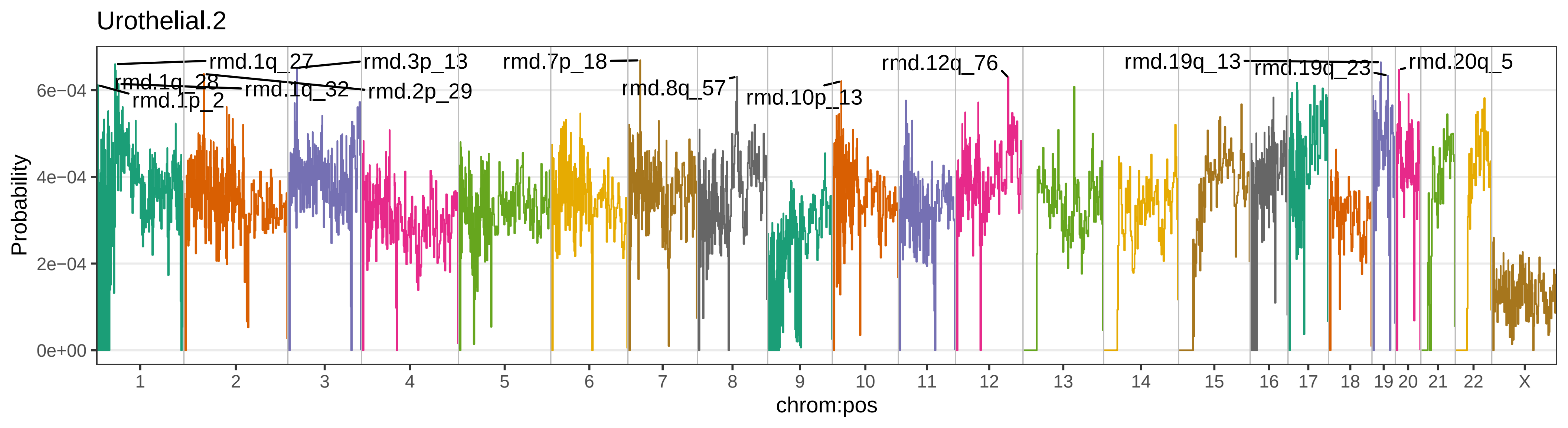

Urothelial.2
rmd.1q_27
rmd.3p_13
rmd.7p_18
rmd.12q_76
rmd.19q_13
rmd.19q_23
rmd.20q_5
rmd.1q_28
rmd.1p_2
rmd.1q_32
rmd.2p_29
rmd.8q_57
rmd.10p_13
Probability
6e−04
4e−04
2e−04
0e+00
1 2 3 4 5 6 7 8 9 10 11 12 13 14 15 16 17 18 19 20 21 22 X
chrom:pos

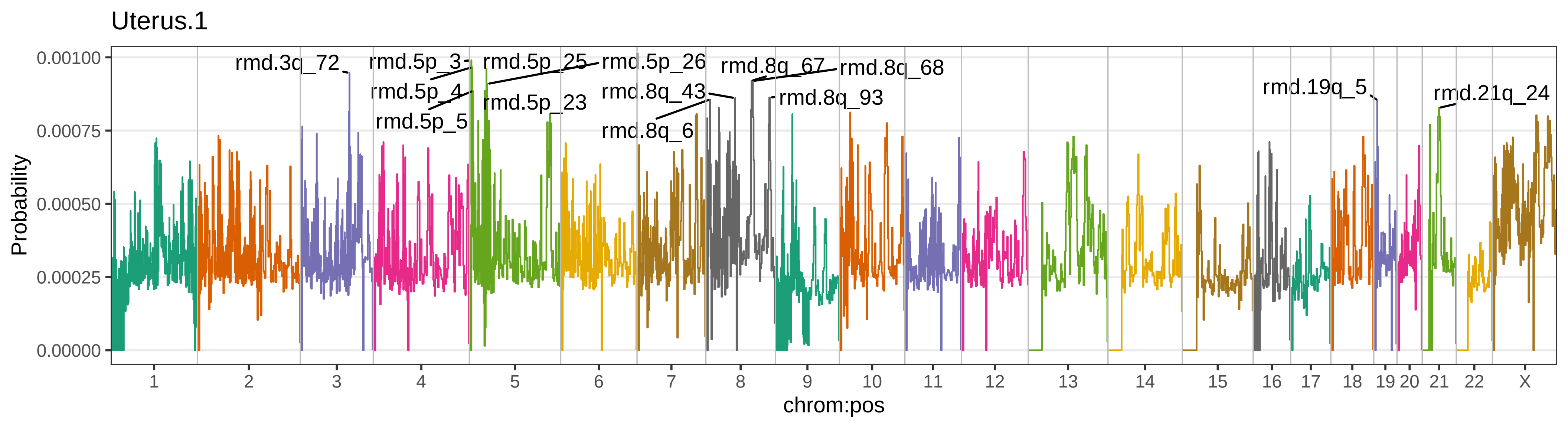

Uterus.1
Probability
0.00100
0.00075
0.00050
0.00025
0.00000
rmd.3q_72
rmd.5p_3
rmd.5p_4
rmd.5p_5
rmd.5p_25
rmd.5p_23
rmd.5p_26
rmd.8q_43
rmd.8q_6
rmd.8q_67
rmd.8q_68
rmd.8q_93
rmd.19q_5
rmd.21q_24
1
2
3
4
5
6
7
8
9
10
11
12
13
14
15
16
17
18
19
20
21
22
X
chrom:pos
